# Taxonomic distribution and origins of the extended LHC (light-harvesting complex) antenna protein superfamily

**DOI:** 10.1186/1471-2148-10-233

**Published:** 2010-07-30

**Authors:** Johannes Engelken, Henner Brinkmann, Iwona Adamska

**Affiliations:** 1 Department of Biology, University of Konstanz, Universitätsstrasse 10, DE-78457 Konstanz, Germany; 2 Institute of Evolutionary Biology (CSIC-UPF), Pompeu Fabra University, Dr. Aiguader 88, 08003 Barcelona, Spain; 3 Centre Robert Cedergren, Département de Biochimie, Université de Montréal, 2900 Boulevard Edouard-Montpetit, Montréal, Québec, H3T1J4, Canada

## Abstract

**Background:**

The extended light-harvesting complex (LHC) protein superfamily is a centerpiece of eukaryotic photosynthesis, comprising the LHC family and several families involved in photoprotection, like the LHC-like and the photosystem II subunit S (PSBS). The evolution of this complex superfamily has long remained elusive, partially due to previously missing families.

**Results:**

In this study we present a meticulous search for LHC-like sequences in public genome and expressed sequence tag databases covering twelve representative photosynthetic eukaryotes from the three primary lineages of plants (Plantae): glaucophytes, red algae and green plants (Viridiplantae). By introducing a coherent classification of the different protein families based on both, hidden Markov model analyses and structural predictions, numerous new LHC-like sequences were identified and several new families were described, including the red lineage chlorophyll *a/b*-binding-like protein (RedCAP) family from red algae and diatoms. The test of alternative topologies of sequences of the highly conserved chlorophyll-binding core structure of LHC and PSBS proteins significantly supports the independent origins of LHC and PSBS families via two unrelated internal gene duplication events. This result was confirmed by the application of cluster likelihood mapping.

**Conclusions:**

The independent evolution of LHC and PSBS families is supported by strong phylogenetic evidence. In addition, a possible origin of LHC and PSBS families from different homologous members of the stress-enhanced protein subfamily, a diverse and anciently paralogous group of two-helix proteins, seems likely. The new hypothesis for the evolution of the extended LHC protein superfamily proposed here is in agreement with the character evolution analysis that incorporates the distribution of families and subfamilies across taxonomic lineages. Intriguingly, stress-enhanced proteins, which are universally found in the genomes of green plants, red algae, glaucophytes and in diatoms with complex plastids, could represent an important and previously missing link in the evolution of the extended LHC protein superfamily.

## Background

The evolution of algae and land plants and their photosynthetic machineries is intimately linked to the extended light-harvesting complex (LHC) protein superfamily. A chlorophyll-binding (CB) motif that is part of a transmembrane (TM) alpha-helix located in the thylakoid membrane is the homologous core structure of this protein superfamily. Several families belong to the extended LHC protein superfamily [1-5], including the LHC proteins, the LHC-like proteins, the subunit S of photosystem II (PSBS), the ferrochelatase II and a new family described in this work, the red lineage chlorophyll *a/b*-binding (CAB)-like proteins (RedCAP). Ferrochelatases are enzymes that catalyze the terminal step in the heme biosynthesis. Two different ferrochelatases exist in plants [6], but since only one of them possesses a CB motif and is imported into chloroplasts [7] we included only ferrochelatase II into our study. Non-homologous pigment-binding proteins, such as the prochlorophyte CB protein family [8], are not considered here. While the PSBS family consists of four-helix proteins [9], the LHC-like protein family is divided into three-helix early light-induced proteins (ELIPs) [2,3], two-helix stress-enhanced proteins (SEPs) [10] and one-helix proteins (OHPs) [11,12], which in cyanobacteria are also called high light-induced proteins (HLIPs) or small CB-like proteins [13,14]. In contrast to LHC proteins, whose primary function is the absorption of light through chlorophyll excitation and transfer of absorbed energy to photochemical reaction centers, members of LHC-like and PSBS families are likely involved in stress protection [2,3,14,15].

Many different models have been proposed for the evolution of the extended LHC protein superfamily [1,2,5,16-19]. Most of them postulate a four-helix intermediate, similar to PSBS, as the ancestor of the LHC, LHC-like and PSBS families, or alternatively, a direct origin from HLIPs [4]. Currently, the interpretation of the function and taxonomic distribution of these proteins is hampered by the absence of clearly defined families and a consistent framework of their evolution.

In the attempt to solve this problem, we systematically searched representative genomic and expressed sequence tag (EST) databases for members of the extended LHC protein superfamily with a focus on LHC-like sequences. Systematic analysis of their taxonomic distribution together with their primary and predicted secondary structures allowed us to provide a coherent classification and to propose an improved hypothesis for the evolution of this superfamily.

## Results and Discussion

### Classification of the extended LHC protein superfamily

**Figure 1:**
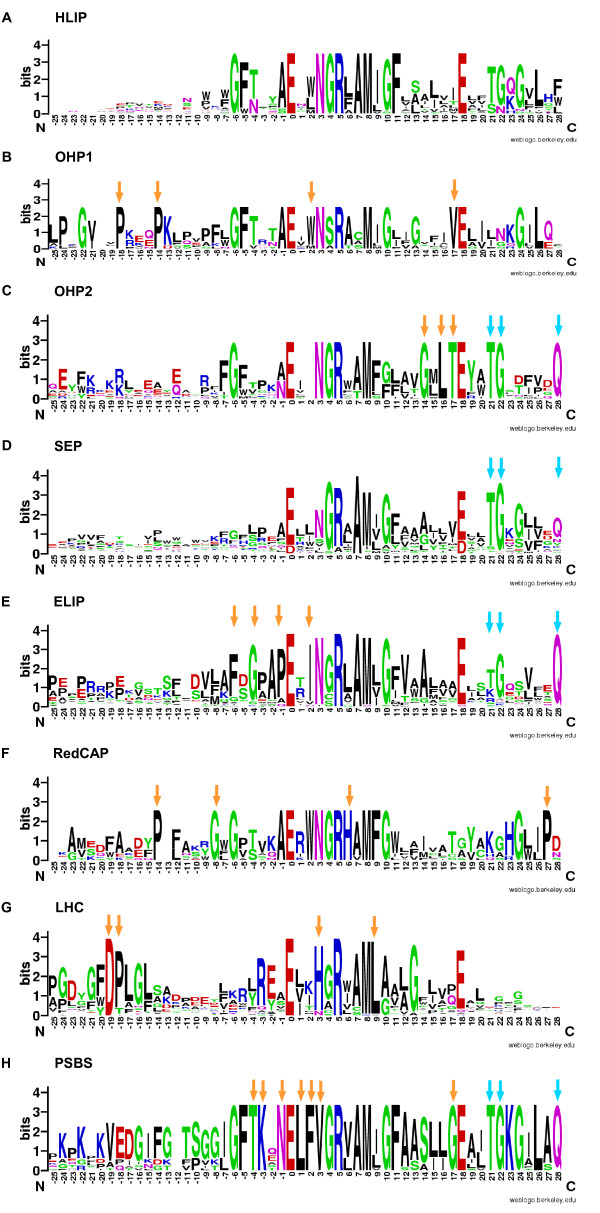
**Sequence logos of representative members of the extended LHC protein superfamily**. (A-H) Sequence logos based on 54 amino acid positions surrounding the CB motif in the first helix were prepared from the same alignments that were used to build the HMM profiles. HMM analysis served as one of several criteria in the classification of unknown sequences (see Methods). The center of the x-axis corresponds to the highly conserved glutamate (E-0). For clarity, the sequence logo associated with the HMM profile of HLIPa is shown for HLIP and for the different SEP HMM profiles, the sequence logo of the combined SEP sequences is shown. An orange arrow marks amino acid residues that are specific to a particular family. Note that threonine (T-21), glycine (G-22) and glutamine (Q-28) are conserved in most SEPs, OHP2, ELIPs and PSBS (marked by a blue arrow). The new RedCAP family is described in more detail in the main text.

We developed a classification scheme for all major families of the extended LHC protein superfamily, based on (i) sequence similarity (hidden Markov model, HMM analysis and BLAST search against a local collection of LHC-like sequences), (ii) secondary structure prediction, and (iii) sequence motifs, like the CB- and carotenoid-binding motifs ([20], see also Methods). Especially the HMM analyses were very powerful in assigning meaningful families. In this way we were able to assign all identified sequences to families and subfamilies (Additional file 1, Table S1). In contrast to HMM analysis, BLAST searches against public databases were not very useful for the purpose of classification due to the often poor or even misleading annotations of the deposited sequences. In order to visualize the HMM analysis as one of several classification criteria, we prepared sequence logos for the common protein families based on the alignments used for the HMM profiles (Figure 1). In order to allow comparison across one-, two-, three- and four-helix protein architectures, these alignments included the conserved CB motifs from the first TM helices.

For each protein family, the HMM profiles captured unique similarity patterns. Interestingly, the sequence logo-plots showed specific and highly conserved amino acid positions for given LHC, PSBS, RedCAP protein families and LHC-like protein subfamilies (marked by orange arrows in Figure 1), in addition to the ubiquitously conserved positions, like glutamate E-0 and arginine R-5. Due to their conservation pattern, these family-specific amino acid residues are expected to be functionally relevant and are likely correlated to specific molecular and physiological functions of the respective protein families. For example, several proline (P) residues are conserved at different positions in OHP1 and the RedCAP family (see orange arrows). Likewise, OHP2, ELIP, LHC and PSBS proteins all possess several specific and highly conserved residues of currently unknown function. In contrast to this, the sequence logos of the LHC-like protein subfamilies, such as HLIP and SEP, do not reveal uniquely conserved amino acid positions, they can, however, be found in subsets of HLIPs and SEPs (data not shown). The most likely explanation is that these two subfamilies are anciently paralogous, in addition, they are the oldest subfamilies. Another set of amino acid positions of potential functional interest are residues that are conserved across a distinct subset of protein families, like several amino acids within the first TM helix and one (glutamine Q-28) at the C-terminal end of the first TM helix in SEPs, OHP2, ELIPs and PSBS (blue arrows in Figure 1).

**Figure 2:**
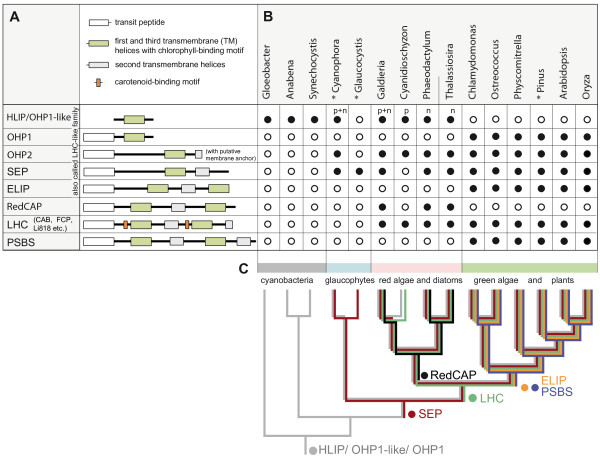
**Schematic overview, taxonomic distribution and ancestral character analysis of the extended LHC protein superfamily**. (A) Schematic overview of the major protein families. The drawing shows the approximate position and relative length of conserved regions and sequence motifs including the CB motif-containing first and third TM alpha helices. Details for each of the identified sequences are given in Additional file 1, Table S1. The classification into a limited set of families and subfamilies was based on three main criteria: (i) sequence similarity to members of already described or newly defined families or subfamilies (like OHP2 and RedCAP) using HMM and local BLAST analysis against our own database, (ii) the predicted secondary structures with the number and order of predicted TM helices, and (iii) predicted sequence motifs, like the CB motifs and carotenoid-binding motifs. One-helix sequences are divided into the well conserved, nuclear-encoded OHP1 limited to the green lineage and the more diverse, plastid-encoded or cyanobacterial HLIPs. Nuclear one-helix sequences with no pronounced similarity to OHP1 are referred to as OHP1-like. (B) Presence (black circle) and absence (white circle) of the LHC superfamily sequences in genomic and EST (indicated by a star*) databases of twelve photosynthetic eukaryotes representing all major lineages of Plantae and three divergent cyanobacteria (both unicellular, and filamentous with heterocysts). Genus names are used (for species names refer to Additional file 1, Table S1). The locations of the genes are marked with "p" for plastid-encoded or with "n" for nuclear-encoded. (C) Ancestral character evolution analysis for plastid-related genes of cyanobacterial origin (see also Methods). The distribution of distinct families of the extended LHC protein superfamily is indicated on a given species tree corresponding to a consensus plastid phylogeny. *Gloeobacter violaceus *was used to root the tree. This analysis suggests an evolutionary origin for the different families, which is indicated on the tree by a colored circle followed by their names.

In order to further visualize the classification scheme, we show a sequence as an example for each of these families (Additional file 1, Figure S1). An example for the classification process can be found in Additional file 1, Table S2 based on the sequences identified in *Cyanophora paradoxa*, a well-studied glaucophyte. The Additional file 1, Table S2 includes assigned accession numbers from the GenBank database, the predicted number of TM helices, the best p-values and scores from the HMM analysis, as well as from the local BLASTP analysis and a suggestion for their classification. An overview of the basic structural elements of members of the extended LHC protein superfamily is shown in Figure 2A.

### Taxonomic distribution

A diverse set of sequences with the characteristic CB sequence motif was found in systematic database searches. The 15 organisms under study represent the three major lineages of Plantae *(Archaeplastida)*, including two glaucophytes, two red algae, two green algae and four divergent land plants (a moss, a conifer, a monocot and a dicot), as well as two diatoms (stramenopiles) and three divergent cyanobacteria. Individual sequences are listed in Additional file 1, Table S1. The sensitivity of our search approach was demonstrated by the identification of numerous previously unreported sequences that belong to the extended LHC protein superfamily, including three sequences from the well-annotated genome of *Arabidopsis thaliana*. Homologous sequences were exclusively found in photosynthetic organisms and were neither present in the ciliates *Tetrahymena thermophila *and *Paramecium tetraurelia *nor in the oomycete stramenopile *Phytophtora ramorum *(related to diatoms) [21], which were recently suggested to have had a photosynthetic ancestry [22]. The only exceptions to this rule are transducing cyanophages (bacteriophages that infect cyanobacteria) that contained several HLIP sequences in their genomes [23].

The identified sequences of the extended LHC protein superfamily show a unique distribution across the taxonomic lineages. The presence/absence of LHC, RedCAP and PSBS families and several LHC-like subfamilies is presented in Figure 2B. An ancestral character analysis within the framework of an established consensus plastid phylogeny suggests the likely order of emergence of the different protein families (Figure 2C, see also Methods). Since the HLIP/OHP1 are ubiquitously distributed among eukaryotes and represent the only group (except for the fusion protein ferrochelatase II) that is also present in cyanobacteria, the cyanobacterial HLIPs are generally assumed the origin of the eukaryotic LHC protein superfamily. They are still plastid-encoded in glaucophytes and red algae. The plastid-encoded eukaryotic HLIPs are orthologs of the nuclear-encoded OHP1 sequences in the green lineage (Viridiplantae), which were transferred to the nucleus via endosymbiotic gene transfer [11], and apparently plastid-encoded HLIPs were lost in the green lineage. Some nuclear-encoded one-helix sequences from the red lineage and glaucophytes were named OHP1-like, but they showed no specific sequence similarity to OHP1. OHP2 are distributed ubiquitously across photosynthetic eukaryotes and are different from HLIP/OHP1 by possessing a short C-terminal hydrophobic element, which is possibly embedded in the thylakoid membrane [12]. In addition, their significantly different primary sequence structure (Figure 1) makes them a unique group within the LHC-like family.

At least some LHC subfamilies, like CAB, fucoxanthin chlorophyll *a/c*-binding proteins (FCPs) and LI818, are present in all major red and green lineages, but apparently not in glaucophytes (Figure 2B). It seems highly unlikely that these abundant proteins would remain undetected in all EST approaches and thus would escape detection in the current study. This absence of molecular data is supported by immunological methods [24]. A 28 kDa protein cross-reacting with an antibody raised against FCP of a marine raphidophyte was reported in glaucophytes [25]. Unfortunately, the question whether this 28 kDa protein belongs to the LHC family remained unresolved since it cannot be excluded that it only shares epitopes with LHC proteins but is structurally different [25]. Based on these studies Koziol and colleagues [19] proposed an origin of LHC proteins at the basis of the green and red algal lineage that is in agreement with our conclusions.

### RedCAPs and other new sequences

While most families of the extended LHC protein superfamily had been described earlier, the nuclear-encoded RedCAPs from the red lineage (Sturm S, Engelken J, Gruber A, Vugrines S, Adamska I, Kroth P, Lavaud J, unpublished) have not been defined yet. The RedCAP family and the OHP2 subfamily can be reliably assigned based on HMM and BLASTP analyses (Additional file 1, Table S1). RedCAP sequences form a well-conserved family, and in contrast to ELIP or LHC proteins also their second helix is conserved. In public databases, RedCAP sequences sometimes are erroneously described as HV60 (based on the name of an ELIP sequence from *Hordeum vulgare*). However, based on primary sequence similarity, sequence length, conservation patterns, HMM analyses and phylogenetic analyses there is no indication that RedCAPs were specifically related to any other group of the extended LHC protein superfamily (Sturm S, Engelken J, Gruber A, Vugrines S, Adamska I, Kroth P, Lavaud J, unpublished). In contrast to the almost ubiquitous OHP2 and SEPs, the RedCAPs are clearly restricted to the red lineage, whereas PSBS and ELIPs are limited to the green lineage without any overlap (Figure 2B and 2C).

In addition to two copies of the already described PSBS in the green alga *Chlamydomonas reinhardtii *[JGI_Chlre4: 196341 and 171516], we identified a third, rather divergent PSBS sequence, which we named PSBS-like [JGI_Chlre4: 175221]. Based on HMM analysis, BLASTP, phylogenetic analysis (data not shown) and the number of TM helices it can be clearly classified as a PSBS (or a PSBS-like) sequence (Additional file 1, Table S1). This new sequence encodes a 311 amino acid long protein that has a highly similar counterpart in *Volvox carteri *with a length of 316 amino acid [JGI_Volca1: 94261]. Both PSB-like sequences are likely functional, based on the highly conserved exonic sequences in the *C. reinhardtii *- *V. carteri *comparison, although no EST is available. As a side-note, it has recently been shown that in *C. reinhardtii*, the common PSBS protein may not be translated under many growth conditions [26]. Notably, a high number of LHC-like sequences were identified in the genome of *Physcomitrella patens *that had partially been described in the general genome analysis [27] and in a second analysis with a special focus on the antenna gene supplement [28].

### Broad taxonomic distribution of the two-helix SEP subfamily

SEPs were defined as a LHC-like subfamily with one characteristic CB motif and a conserved secondary structure with two TM helices. SEPs are absent in cyanobacteria, but they seem ubiquitously distributed in photosynthetic eukaryotes. A total of 40 SEP sequences were identified in 15 organisms. In the glaucophytes we found six sequences in *Glaucocystis nostochinearum *and two in *C. paradoxa *and in streptophytes, six sequences in *A. thaliana *and with nine the largest number in the moss *P. patens *(Additional file 1, Table S1). Among the Cyanidiales, the red alga *Galdieria sulphuraria *has one SEP, whereas the completely sequenced thermo- and extremophile red alga *Cyanidioschyzon merolae *has none. In algae with complex plastids, we have detected a single rather divergent SEP in each of the two diatoms *Phaeodactylum tricornutum *and *Thalassiosira pseudonana *and in the pelagophyte *Aureococcus anophagefferens*. The presence of only one SEP in these taxa could be due to a streamlining process of the genome size of these particular taxa. Therefore, SEPs do not appear to be essential in the red lineage and seem to have been secondarily lost from several taxa.

**Figure 3:**
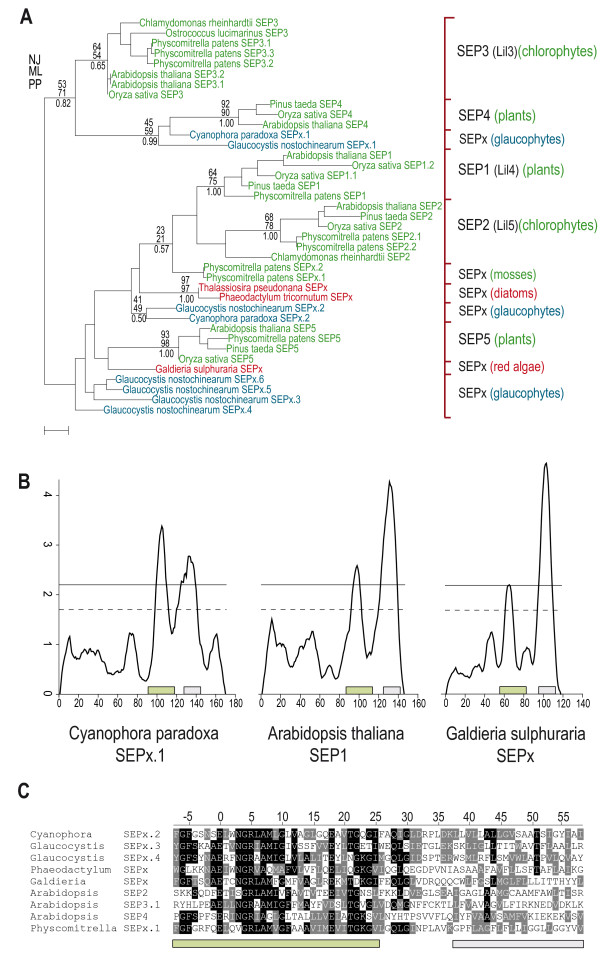
**Phylogeny, predicted primary and secondary structures of two-helix SEPs**. (A) Phylogenetic analysis including all 39 identified SEP sequences (except the partial SEP3/Lil3 from *Pinus taeda*) from twelve photosynthetic eukaryotes. The maximum likelihood tree was inferred from 32 amino acid positions, only bootstrap values at nodes supported by a posterior probability of ≥0.50 are given. 10,000 bootstrap replicates using the Dayhoff+Γ4 model to estimate the pairwise distances in the neighbor-joining analysis with MEGA4; 100 replicates in the maximum likelihood analysis with PhyML, 3 million generations with 1/3 discarded (burn-in) in the Bayesian analysis to estimate the posterior probability with MrBayes. The probabilistic methods were using the WAG+Γ4 model with four discrete gamma rate categories. This tree gives an overview of the diversity of the SEP sequences within all major lineages of Plantae. Blue, red and green colors indicate glaucophytes, red algae and algae with complex plastids, as well as green algae and land plants, respectively. This analysis is compatible with the assumption of several ancient, paralogous groups within the SEP subfamily. SEPs from red algae and SEP4 and SEP5 from land plants are reported for the first time. (B) Prediction of TM alpha helices in SEP sequences from a glaucophyte, a land plant and a red alga. The first of the two predicted TM helices comprises the CB motif. (C) Alignment of typical SEP sequences from all three major lineages of Plantae. The approximate positions of the predicted first and second TM helices are underlined with a green and a grey bar, respectively. Identical and similar amino acids are shown in white on black and grey backgrounds, respectively.

Phylogenetic analysis of the SEP sequences shown in Figure 3A suggests the presence of several ancient paralogous groups, with the highest diversity found in land plants containing five SEP1-5 groups. Notably, two previously not described orphan SEP groups, SEP4 and SEP5, were found in land plants. SEP groups other than SEP1-5, despite some affinities based on HMM and phylogenetic analysis, were preliminarily named SEPx. SEPx.1 and SEPx.2 form two of several ancient SEP lineages in glaucophytes and both orthologs are present in two rather distantly related species, *C. paradoxa *and *G. nostochinearum*. Diatoms have similar to red alga a low number of SEP sequences. The phylogenetic tree is compatible with the assumption of several ancient paralogous groups within the SEP subfamily. However, the question whether all SEPs are monophyletic or if certain groups, like the highly conserved SEP3 (Lil3), emerged later independently, remains an open question. Also the ultimate number of groups within the SEP subfamily is not known and an answer will surely have to await at least until many more completely sequenced and taxonomic diverse genomes are available. Although, there is currently no solid support, neither for nor against the monophyly of SEPs, a single origin seems the most parsimonious scenario. We note that divergent groups, like SEP3 (Lil3), branch more solidly with other SEPs within the subfamily, when more (and previously missing) SEP groups are being added to the phylogenetic analysis (Figure 3A).

The similarity in predicted secondary structure of SEPs is shown for selected SEP sequences from the three major lineages of Plantae, i.e. glaucophytes (*C. paradoxa*), red lineage (*G. sulphuraria*) and green lineage (*A. thaliana*) in Figure 3B, using the Dense Surface Alignment (DAS) algorithm [29]. The high similarity of their predicted primary structure is displayed in Figure 3C. In cyanobacteria and cyanophages, no SEP-like sequences have been found, which is consistent with the idea of a eukaryotic origin of SEPs. The recent finding of a fusion protein with two predicted TM helices in a *Synechococcus *strain, termed hli5OS-B' (similar to YP_478210) [30], is not relevant for our study, since the order of the two TM helices ("coh1" precedes the CB motif), is inverted compared to two-helix SEPs.

The identification of SEPs, as well as OHP2, in the red lineage and in glaucophytes (Figure 2B and 2C and Additional file 1, Table S1) is a notable extension of their previously known distribution within chlorophytes and streptophytes (Viridaeplantae). This distribution argues strongly in favor of an early origin of SEPs and OHP2 in the common eukaryotic ancestor of Plantae that predates the origin of all three- and four-helix proteins (Figure 2C).

### Independent origins of LHC and PSBS families in discrete duplication events

The PSBS proteins are predicted to form four-helix structures that were proposed to have originated in internal gene duplication [31,32]. It was also noted, that the two halves of PSBS are more related to each other than the comparable parts of the LHC protein [16]. In contrast to PSBS, the origin of LHC proteins is less clear. Among other scenarios [4], it was suggested [16] that LHC and PSBS proteins evolved from a common four-helix ancestor (Additional file 1, Figure S2).

**Figure 4:**
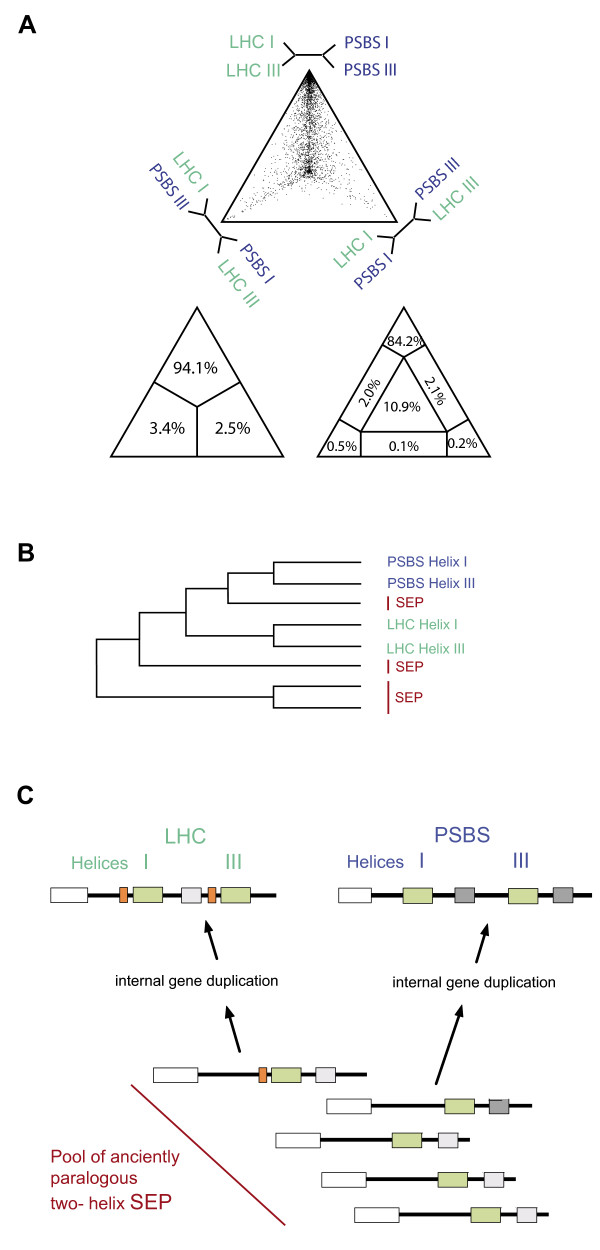
**Phylogenetic analysis and the independent origins of PSBS and LHC families**. (A) Four-cluster likelihood mapping analysis [33] of individual CB motifs in LHC and PSBS proteins showing the likelihood support for three alternative topologies. 64 amino acid positions (32 per CB motif) from 60 protein sequences including all known LHC subfamilies [19] and the complete PSBS diversity were analyzed. The topology with LHC helix I as sister group to LHC helix III is strongly supported with 94.1% over the one expected under a common origin with 2.5% in the three-partite diagram (or 84.2% over 0.2% in the seven-partite diagram). (B) Schematic diagram of a phylogenetic analysis based on the conserved sequence motifs of the first and third TM helices of PSBS and LHC proteins. A selection of 22 diverse SEP sequences from glaucophytes, red algae and the green lineage was included. The alignment contained 31 unambiguously aligned amino acid positions. The distinct clustering of the different LHC and PSBS helices provides corroborating evidence that LHC sequences do not share a common four-helix ancestor with PSBS sequences. The shown topology was significantly (p = 0.0001) supported over the alternative topology (Additional file 1, Figure S1B), analyses were done in TreePuzzle. The true tree is presented in Additional file 1, Figure S2. (C) Hypothesis for the independent origin of LHC and PSBS proteins from distinct SEP ancestors according to the present study. LHC proteins likely evolved from a SEP with a putative carotenoid-binding motif (orange box), which was duplicated together with the CB motif in an internal gene duplication/unequal crossing-over of tandem genes. PSBS protein evolved from a different ancestor from an ancient pool of paralogous SEP members. PSBS protein has highly conserved second and fourth helices (dark grey boxes). Note that following this hypothesis, helices I and III of the resulting LHC and PSBS proteins must be most similar within the same proteins, which is in agreement with (A) and (B) but in conflict with a previously suggested scenario (Additional file 1, Figure S1).

However, our phylogenetic analyses (Figure 4 and Additional file 1, Figure S3) do not support this scenario but strongly favor two independent duplication events. In order to evaluate the evidence for or against a common origin of LHC and PSBS families, a phylogenetic analysis of individual CB-TM helices from a representative and large number of different LHC and PSBS sequences was performed. Therefore, 10,000 random puzzle quartets created out of the 120 individual helices of 32 amino acid length (originally extracted out of 60 protein sequences with 64 aligned amino acid positions), corresponding to the first and third helices of both, LHC and PSBS proteins, were mapped on three possible topologies (Figure 4A, top triangle) in a four-cluster likelihood mapping analysis ([33], see also Methods).

In the tri-partite diagram, 94.1% of the quartets support the respective sister-group relationship of helices I and III between both, LHC and PSBS proteins, versus 2.5% support the topology expected under the scenario of a common origin. In the more stringent diagram (Figure 4A, right bottom triangle divided into seven areas), the result was 84.2% versus 0.2% and 0.5%, with 10.9% that are not in favor of any of the three alternative topologies (unresolved quartets). This result strongly contradicts the often favored, long-standing evolutionary scenario of a common origin shown in Additional file 1, Figure S2A [16]. In this case the first and the third helices of both, LHC and PSBS proteins, would most resemble each other and therefore cluster together in a phylogenetic tree. This tree topology is displayed at the bottom right corner of the PUZZLE triangle diagram of Figure 4A and should be the only one supported. However, this alternative is supported by only 2.5% and 0.2%, respectively. The support for the common origin is therefore even lower than the support for the biologically unrealistic solution of a hybrid LHC/PSBS protein that is nevertheless supported by 3.4% and 0.5%, respectively (Figure 4A, left bottom triangle).

We accounted for the high degree of sequence diversity in the complex LHC protein family by incorporating all five LHC subfamilies (CAB, Li818 and Li818-like, the red algae/cryptomonad LHC and FCP), as well as a newly described clade LHCz [19]. PSBS likewise were chosen from taxonomically distant green algae and land plants. When the dataset was reduced by removing the fast-evolving (long-branch) *Ostreococcus tauri *PSBS and the PSBS-like sequences from *V. carteri *and *C*. *reinhardtii*, as well as the divergent LHCz and FCP sequences, the percentage of unresolved quartets strongly decreased and the support for the clustering of helices I and III was further improved (99.8% versus 0.2%, Additional file 1, Figure S4A). However, to include the maximal sequence diversity we show the more conservative result of the larger dataset with 120 sequences (Figure 4A). The presence of a great number of phylogenetic diverse sequences resulted in a highly informative alignment, despite its short length of 32 amino acid positions. Sequences could be readily aligned due to the virtual absence of both gaps and insertions within and surrounding the CB-TM helices. We chose to limit the analysis to a short but accurate alignment and avoided the potentially dangerous inclusion of many unreliably aligned positions. Nevertheless, the result is quite robust to the inclusion of more noisy positions (data not shown), but note the major difference observed after the removal of fast evolving and divergent sequences (84.2% versus 99.8%). The surprisingly strong phylogenetic signal is also reflected in a low percentage of partially (4.2%) and completely (10.9%) unresolved quartets in this analysis (Figure 4A), which are essentially due to the inclusion of divergent primary sequences.

In the attempt to further validate our finding we inferred a maximum likelihood tree using a representative set of CB-TM sequences from LHC and PSBS families and the SEP subfamily (shown schematically in Figure 4B and entirely in Additional file 1, Figure S3). The results revealed that helices I and III of PSBS protein formed a monophyletic group (bootstrap value 85/83, with and without gamma correction) and the same situation was encountered for the LHC helices, albeit with weaker support (bootstrap value 51/41). To test if this topology (Figure 4B) was significantly better than the one expected under the old scenario (Additional file 1, Figure S2B), the expected likelihood weight and the Shimodaira-Hasegawa topology tests were performed in Tree-Puzzle. The scenario of Figure 4B is supported at a very high significance level (p = 0.0001) by both tests. In agreement with this result, we note that the duplicated area within both LHC and PSBS sequences extends substantially beyond the shared CB-TM helices and is not homologous between the two groups.

Functional constraints acting on CB motifs could hypothetically interfere with the genuine phylogenetic signal analyzed. However, this should affect functional sites and these sites (like glutamate E+0, histidine/asparagines H/N+3 or arginine R+5 in LHCII from spinach *Spinacia oleracea*, [34]) are conserved to such a high degree that they essentially do not contribute to the phylogenetic signal. This was confirmed in an additional likelihood mapping analysis, where these three functional sites were omitted (89.3% versus 5.0%, Additional file 1, Figure S4B). Furthermore, the CB motifs are generally under purifying selection maintaining structure and function. This selective force results in divergent rather than convergent evolution. Therefore, we conclude that potential functional constraints do not substantially interfere with the phylogenetic signal.

### Character evolution and the possible origins of LHC and PSBS families from distinct SEPs

Since all LHC subfamilies share a common origin [35] and are absent in glaucophytes, the first LHC proteins did most likely evolve in a common ancestor of the red and green lineages ([19] and Figure 2C). The presence of two-helix SEPs in all three lineages of plants and deduced from this distribution their existence in the common ancestor, potentially already in form of paralogous copies (Figure 2C), make them prime candidates for the origin of LHC proteins. The internal gene duplication of a two-helix sequence would provide a simple and parsimonious explanation for the origin of the second, less-conserved CB-TM helix in LHC proteins. This makes SEPs a better candidate for the origin of LHC proteins than the previously proposed HLIPs [4,19]. Furthermore, in eukaryotes HLIPs tend to occur as single copy genes and are plastid-encoded, whereas the internal gene duplication/unequal crossing-over event of tandem genes from which the first LHC protein evolved, is very likely to have taken place in the nuclear genome. There are several different processes, which may result in an internal gene duplication: (i) a slippage of the replication apparatus may lead either to a duplicated or to a deleted region, this process is happening rather frequently and is leading to duplicated areas (genes) arranged in tandem, and/or (ii) if there are already at least two closely related copies of a gene arranged in tandem, an unequal crossing-over between different copies on the two sister-chromosomes may lead to a fusion of parts of two genes (resulting in an internal duplication) on one chromosome and to a truncated copy on the other.

In light of these mechanistic considerations it seems that genes, which (i) occur in tandem repeat units, and (ii) are nuclear-encoded did most likely provide the genomic context for the proposed internal gene duplication. In addition, even a nuclear copy of a HLIP/OHP arranged in tandem would not be sufficient to generate a LHC protein, since there are no second transmembrane helices, which would need to be newly created. Again, all these characteristics favor SEPs over HLIPs as candidates for the origin of LHC proteins.

Most members of the LHC family contain the well-conserved carotenoid-binding motif [20] consisting of the amino acid residues FDPLGL (or similar) found approximately 15 amino acid positions in front of the CB motif in both, the first and third TM helices. However, neither RedCAP and ELIP nor PSPS family members harbor this specific carotenoid-binding motif in any of the two possible locations. This would make a two-helix protein that already contained the carotenoid-binding motif the most likely source for the origin of LHC proteins. Intriguingly, we found a SEP sequence (named here SEPx.4) in the glaucophyte *G. nostochinearum *that contains the three core amino acid residues FDP of the carotenoid-binding motif in the expected distance from the CB motif (Additional file 1, Figure S5).

**Figure 5:**
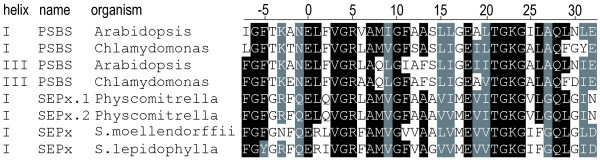
**Alignment of PSBS and a group of selected SEP sequences from early land plants**. Two representative PSBS sequences including only the first (I) and third (III) helices show high similarity to four SEPx sequences from *S. moellendorffii*, *Selaginella lepidophylla *and *P. patens*. Identical amino acids shown in white are surrounded by a black and similar amino acids by a grey box. Note the conserved valine (V) in both groups (position V-3), a potential CB position at which most other protein families carry an asparagine (N-3) or histidine (H-3).

Taken together, analyses of the individual helices including additional and independently conserved elements in LHC and PSBS sequences provide direct evidence for their origins by distinct internal gene duplication events. Likely, from a pool of paralogous two-helix SEPs, one SEP subfamily member gave rise to the LHC protein family by internal gene duplication. Likewise, the PSBS protein family evolved from a distinct SEP (Figure 4C). This view is corroborated both by extensive database searches and a recent study [19] showing that PSBS is widespread within, but restricted to the green lineage. Interestingly, a cluster of newly identified SEP sequences in mosses, e.g. the slowly evolving SEPx from *P. patens *(recently described as Lil7 in [28]), shows rather high sequence similarity to PSBS helices I and III from both *A. thaliana *and *C*. *reinhardtii *(Figure 5) and thus, could represent the ancestral SEP subfamily from which PSBS originated.

### Evaluation of alternative scenarios

Many attempts have been made to solve the question of the order in which the different families and subfamilies of the extended LHC protein superfamily have originated [5,16,19,35,36]. The existence of a LHC-like protein with only two TM helices as the ancestor of three- and four-helix proteins was already predicted more than one decade ago [16,37], but the first experimental proof was only presented many years later in *A. thaliana *[10]. While the number and diversity of identified sequences and families progressively increased, the order of their emergence remained enigmatic. The reason for this major limitation lies in the small size of their defining element, the CB helix, and the considerable age of the families under study, which renders it impossible to simply deduce the order of their emergence from a phylogenetic analysis of the primary sequences. In order to overcome this limitation, we took advantage of (i) the new wealth of sequence data with special emphasis on completely sequenced genomes, (ii) recent multi-gene phylogenies that established a solidly supported phylogenomic tree of plastids, as well as the basal position of glaucophytes [38], and (iii) an independent phylogenetic approach in which we test the hypothesis of independent origins of the LHC and PSBS families.

An initial hypothesis for the order of emergence of the different family members was deduced from their taxonomic distribution using the ancestral character evolution analysis (Figure 2C). Although the "tree of eukaryotes" is still far from being resolved [39], the topology of the underlying (plastid) tree of photosynthetic eukaryotes used in this study is supported by several publications [40-43]. By relying on established group/species relationships, character evolution is independent of the potential pitfalls of phylogenies based on a single or a few genes.

The establishment of a rigorous classification scheme for the various families of the extended LHC protein superfamily was based on primary and secondary sequence information from a comprehensive database search. The fact that independent approaches (BLAST, HMM, different phylogenetic methods, TM helix analysis) led to mutually compatible results make us confident that the proposed relationships reflect to some detail biological realities.

A possible alternative for the origin of certain families could be to assume their origin at an earlier stage, e.g. the PSBS in the common ancestor of red/green lineages. However, apart from requiring a complete secondary loss in several lineages, this scenario would not provide more plausible explanations for the origin of the remaining families. Individual families, nevertheless, may have experienced isolated losses in certain taxonomic groups, like the SEPs that were lost in the extremophile red alga *C. merolae *and in certain algae with complex plastids, e.g. *Emiliania huxleyi*, but not in *G. sulphuraria*, in diatoms or in the pelagophyte *A. anophagefferens*. A broad taxonomic distribution, the presence of CB motifs and a conserved secondary structure would support a role of SEPs as recurrent building blocks of three-helix proteins, like LHC and four-helix proteins, like PSBS.

Based only on EST databases we cannot rule out the presence of additional relevant protein families of the extended LHC protein superfamily, for example in the two glaucophytes. However, we note that the chosen databases present very substantial numbers of unique ESTs. For *C. paradoxa *9,867 unique EST clusters are available at TBestDB [44], which were derived from two different EST libraries, one based on mRNAs from "high light" and the other from "low light regular" conditions. TBEST contains also 4,673 EST clusters derived from *C. paradoxa *grown under different CO_2 _environments, as well as 8,745 unique EST clusters from *G. nostochinearum*. Additional glaucophyte ESTs are available from NCBI. Hence, the risk of overlooking important relevant protein families is substantially reduced due to the availability of different environmental conditions, with the ultimate proof being the genome sequences of these two distantly related glaucophytes. Based on available resources we currently assume that ELIP, RedCAP, LHC and PSBS proteins do not exist in glaucophytes. In addition, even if new LHC-like families were found in glaucophytes, this would not affect the independent origin of LHC and PSBS families.

### Hypothesis for the evolution of the extended LHC protein superfamily

**Figure 6:**
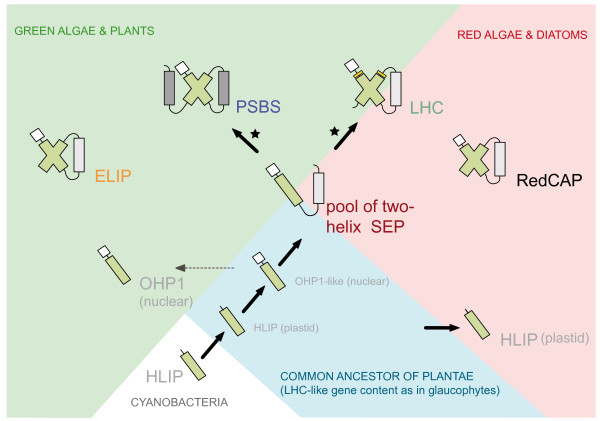
**Proposed scenario for the evolution of the extended LHC protein superfamily**. Originating from a cyanobacterial HLIP after the primary endosymbiosis, an endosymbiotic gene transfer resulted in a nuclear-encoded OHP1-like sequence in the common ancestor of Plantae. Such a sequence was likely at the origin of the nuclear-encoded two-helix SEPs that are ubiquitously distributed among photosynthetic eukaryotes. In Viridiplantae and several algae with complex plastids, the plastid-encoded HLIP was transferred to the nuclear genome and subsequently lost in the plastid. The ancestor of the LHC proteins and later of PSBS evolved by independent, internal gene duplication events (indicated by a star*), likely from different SEP groups. The resulting LHC proteins subsequently either lost their fourth TM helix or alternatively they ancestrally never had one. Both, the ELIPs and the RedCAP family are restricted to the green and to the red lineages, respectively. The positioning of the different family names and the background color indicate their taxonomic distribution.

Similar to previous models, we assume a stepwise evolution from the cyanobacterial HLIPs to the central group of SEPs (Figure 6). However, a crucial novelty of our model is the independent origins of PSBS and LHC families, and possibly also the other three-helix protein families. Based on character evolution analysis (Figure 2C), sequence motifs and the evidence for the independent origins of the PSBS and LHC families (Figure 4) we propose that (i) early LHC proteins originated in the red/green ancestor, likely from a SEP, and subsequently diversified into different antenna proteins in the red and green lineages, and (ii) PSBS arose early in the evolution of the green lineage from a distinct SEP. With less certainty, as mainly based on the taxonomic approaches, we further propose that (iii) ELIPs are neither ancestral to PSBS nor to LHC proteins, but possibly evolved independently in the green lineage, and (iv) RedCAP sequences are limited to the red algal lineage.

The proposed model (Figure 6) can explain the diversity of the extended LHC protein superfamily in cyanobacteria and photosynthetic eukaryotes. Notably, paralogs of the identified two-helix SEPs likely represent an important missing link in the evolution from the ancestral HLIPs to their three- and four-helix descendants in eukaryotes. Furthermore, this model does neither invoke events of horizontal gene transfer nor massive secondary losses, although it requires an additional internal gene duplication event. The discovery of many new and sometimes distantly related sequences (e.g. three in the well-annotated *A. thaliana *genome) suggests that our search has identified all available canonical LHC-like sequences in the surveyed genomes. Additional database searches (see Methods) were in agreement with these results and conclusions.

## Conclusions

Using sequence data from a wide diversity of photosynthetic eukaryotes, cyanobacteria and non-photosynthetic organisms we identified many new members of the extended LHC protein superfamily. We propose a simple and powerful classification scheme based on predicted primary and secondary structures. A new and coherent hypothesis of the evolution of the extended LHC protein superfamily was inferred (Figure 6), supported by comparative genomics and molecular phylogenetic approaches. Importantly, the present study sheds light on the significance of two-helix SEPs and other LHC-like proteins with the discovery of their unexpected diversity and characteristic distribution across photosynthetic eukaryotes. From these evolutionary patterns we expect that proteins of the LHC-like family perform important, yet largely unknown, functions in photoprotection and regulation of photosynthesis.

## Methods

### Sequence search and annotation

Initially, fully sequenced genomes and large EST databases (Additional file 1, Table S1) representing twelve photosynthetic eukaryotes (Plantae) and three cyanobacteria were searched for sequences belonging to the extended LHC protein superfamily. Subsequently, sequence data from additional genomes were collected from public databases including TBestDB [44], NCBI http://www.ncbi.nlm.nih.gov, TIGR http://www.jcvi.org, Kazusa http://bacteria.kazusa.or.jp/cyanobase and UniProt http://www.uniprot.org. We excluded some available genomes from the ancestral character evolution analysis either because of their unclear taxonomic position (*E. huxleyi*), their preliminary nature (*Fragilariopsis cylindrus*, *A. anophagefferens, V. carteri, Chlorella sp., Micromonas sp*. and *Selaginella moellendorffii*), or their highly similar content of LHC-like sequences to *A. thaliana *(*Populus trichocarpa, Arabidopsis lyrata*, *Vitis vinifera*), *Oryza sativa *(*Sorghum bicolor*) or *Ostreococcus lucimarinus *(other *Ostreococcus *spp.) genomes. Database searches were done with the TBLASTN and BLASTP algorithms using consensus sequences for individual subgroups and non-stringent e-values (e = 0.1). When public annotations were unclear or missing, the genes were annotated manually with the helpτmes. Database searches were done with the TBLASTN and BLASTP algorithms using consensus sequences for individual subgroups and non-stringent e-values (e = 0.1). When public annotations were unclear or missing, the genes were annotated manually with the help of the GeneWise algorithm [45] and the tools at the genome browser of the Joint Genome Institute http://genome.jgi-psf.org. EST sequences were translated and manually controlled for frame-shifts that might have created artifacts and gene models were submitted to TPA_inf at NCBI http://www.ncbi.nlm.nih.gov/Genbank/TPA-Inf. For transit peptide prediction the predictor ChloroP was used http://www.expasy.org/tools. In diatoms, the presence/absence of a characteristic N-terminal signal sequence [46] and in red algae the twin-arginine motif [47] were used for signal peptide prediction. All identified LHC-like sequences from 15 organisms are given in Additional file 1, Table S1. Genes with identical deduced amino acid sequence, but different genomic location, as well as closely related ELIPs arranged in tandem (especially in *P. patens*), were listed under a single accession number. In general, annotation was straightforward due to the shortness and intron-scarcity of most LHC-like sequences, as well as due to the presence of transit peptides and signal sequences.

### Classification of sequences

For the HMM analysis [48] we prepared seed alignments containing the first CB motif for each known or newly found protein family. For HLIPs and SEPs, several starting sequences were chosen in order to adequately cover their entire sequence diversity. The length of all alignments was limited to 54 amino acid positions in order to allow the comparison across all families. The seed alignments were augmented in a step-wise manner with the best hits from a sequence search in our local dataset consisting of all identified sequences from 15 organisms and used to create sequence logos (http://weblogo.berkeley.edu, Figure 1). From the same alignments we built a conservative HMM database containing 18 profiles (Additional file 1, Table S1). After calibration we searched the entire local collection of LHC-like sequences from 15 organisms against this HMM database. The three best hits to the local HMM profile database are given in Additional file 1, Table S1. Starting from full-length sequences, we used BLASTP [49] version 2.2.10 to search all sequences against the same local collection of sequences. *P. patens *ELIPs were not numbered due to the number of ELIPs in tandem and therefore, they were not part of the Local Reference Set used for BLASTP. The four best local BLASTP hits against the local collection of LHC-like sequences are given in Additional file 1, Table S1. HMM profiles were the most sensitive tool for classification of LHC-like sequences and this classification was complemented by local BLASTP analysis that have the advantage of using the entire sequence. Prediction of prokaryotic TM alpha-helices was done with the DAS program [29,50] (all proteins were treated as prokaryotic, since they are most likely of cyanobacterial origin and are active in the chloroplast). CB motifs were automatically designated as TM helices due to their experimentally derived helix structure in a LHC protein from photosystem II [34,51].

The most efficient criteria for classification differed slightly among the protein families and depended on their degree of conservation and on the length of conserved sequence domains. The HLIPs are clearly defined by their one-helix structure, together with being plastid-encoded in eukaryotes. HMM analysis, together with the predicted one-helix structure, is sufficient to define the OHP1 subfamily, which is nuclear-encoded after endosymbiotic gene transfer of the HLIPs in green plants. OHP2 are best classified by HMM and local BLASTP analysis due to their well-conserved primary protein structure. SEPs were classified based on the order of their two TM helices (CB motif containing helix precedes a second TM helix) and sequence similarity to other SEPs, as evident from HMM and BLASTP analyses. Subdivision into SEP1-5 was based on HMM, BLASTP and phylogenetic analysis. ELIP sequences were classified based on HMM, BLASTP and their three-helix structure. Accordingly, RedCAP and LHC sequences, including LHC proteins associated with photosystem I (LHCa) and photosystem II (LHCb), FCP, Li818 and LHCz, and the four-helix PSBS were unequivocally classified based on HMM, BLASTP and their predicted number of helices. The two fusion proteins, ferrochelatase II and Rieske-like CAB protein, possess a less-conserved CB motif (which can be missing in some cases) and therefore, the most efficient classification criterion for these two groups was full-length sequence similarity based on BLASTP.

### Character evolution and phylogenetic analysis

Character evolution based on parsimony (unordered model) was used as implemented in Mesquite [52] for the reconstruction of ancestral states. The analyzed taxa were chosen to obtain a good representation of all photosynthetic organisms that possess members of the extended LHC protein superfamily. Amino acid sequence alignments were done with M-Coffee [53] and manually refined in Bioedit [54]. Informative sites for phylogenetic analyses were chosen using G-blocks [55] with manual refinement. Amino acid substitution matrices for the SEP analysis (Figure 3A) were chosen with ProtTest [56]. Neighbor-joining bootstrap values (10,000 replicates) were obtained in MEGA4 [57]. Maximum likelihood bootstrap analyses with 100 replicates were performed using PhyML [58], posterior probabilities were calculated using MrBayes (3 million generations, the first 1 million trees were discarded as "burn-in") [59], the latter two using a WAG+Γ4 model (Figures 3C and Additional file 1, Figure S2). Consensus trees were created with the Consense option of the PHYLIP package [60]. The significance of alternative topologies (Figure 3) was tested in Tree-Puzzle [61] using the Shimodaira-Hasegawa [62] and the expected likelihood weight [63] tests.

The four-cluster likelihood mapping analysis was performed with Tree-Puzzle [61] using the Dayhoff substitution matrix with four discrete gamma distributed categories. An approximate parameter estimation with quartet sampling for the substitution process and rate variation based on a neighbor-joining tree and 10,000 randomly chosen quartets were used. The dataset included a total of 120 sequences, with 41 pairs of LHC and 19 pairs of PSBS (helices I and III) sequences, respectively.

### Accession numbers

Sequence data of newly identified sequences from *C. paradoxa *and *G. nostochinearum *are available in the Third Party Annotation Section of the DDBJ/EMBL/GenBank databases under the accession numbers TPA: BK006744-BK006754. Gene models of all identified sequences from 15 organisms are listed in Additional file 1, Table S1.

## Authors' contributions

JE carried out the database search, JE and HB designed and carried out the analysis of data and drafted the manuscript. IA supervised the project, all authors read and approved the final manuscript.

## Supplementary Material

Additional file 1 **a PDF containing Figures S1-S5 and Table S1 and S2 (**Additional file 1**)**. Click here for file
