# Supplementary material for: Taxonomic distribution and origins of the extended LHC (light-harvesting complex) antenna protein superfamily
Source: BMC Evol Biol. 2010 Jul 30;10:233. doi: 10.1186/1471-2148-10-233 (PMC3020630; doi:10.1186/1471-2148-10-233)
Supplement: Additional file 1 — a PDF containing Figures S1-S5 and Table S1 and S2 (Additional file 1). [file 1471-2148-10-233-S1.PDF]

Supplemental Figures

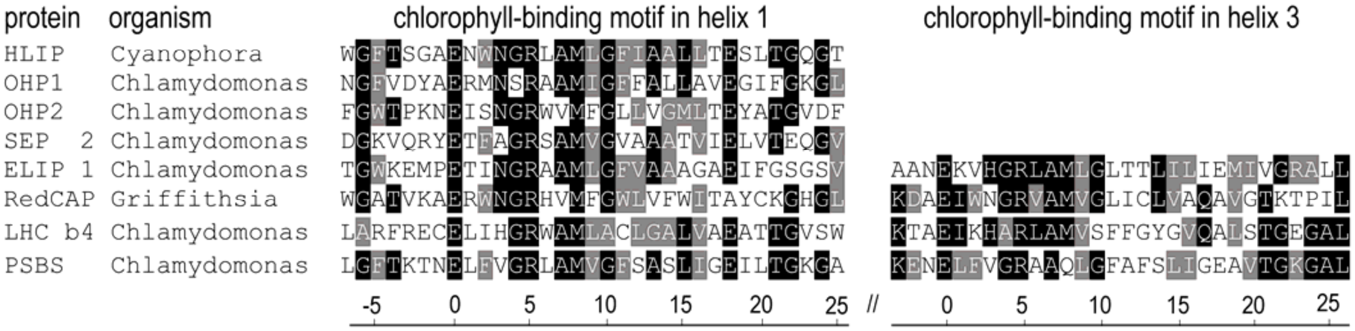

Figure S1

Alignment of conserved sequence motifs within eight representative members from the extended LHC protein superfamily. Accession numbers are listed in Table S1 in Additional data file 1 except RedCAP from *Griffithsia japonica* (Uniprot Q7XZ09). Identical amino acids are shown on a black and similar amino acids on a grey background.

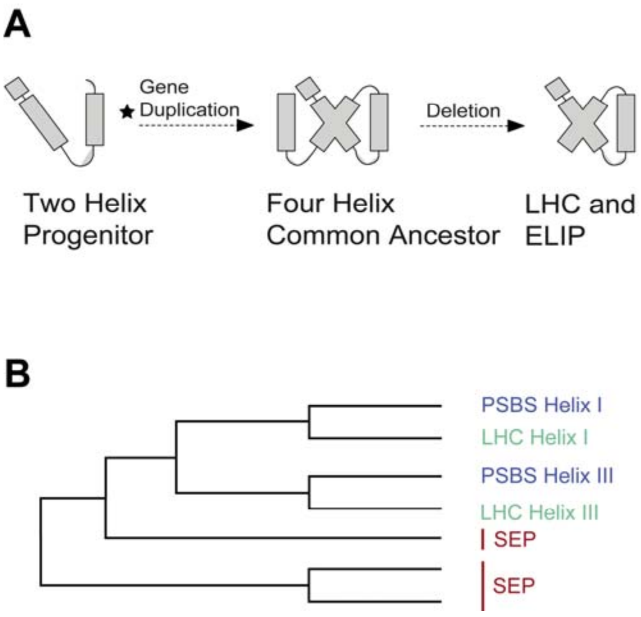

Figure S2

Alternative Origin of LHC and PSBS that is not supported by phylogenetic analysis. (A) The often favored, long-standing evolutionary scenario according to [14]. A two-helix progenitor gave rise to a PSBS-like four-helix common ancestor that evolved into modern three-helix LHC and ELIP sequences. (B) Alternative tree corresponding to this scenario.

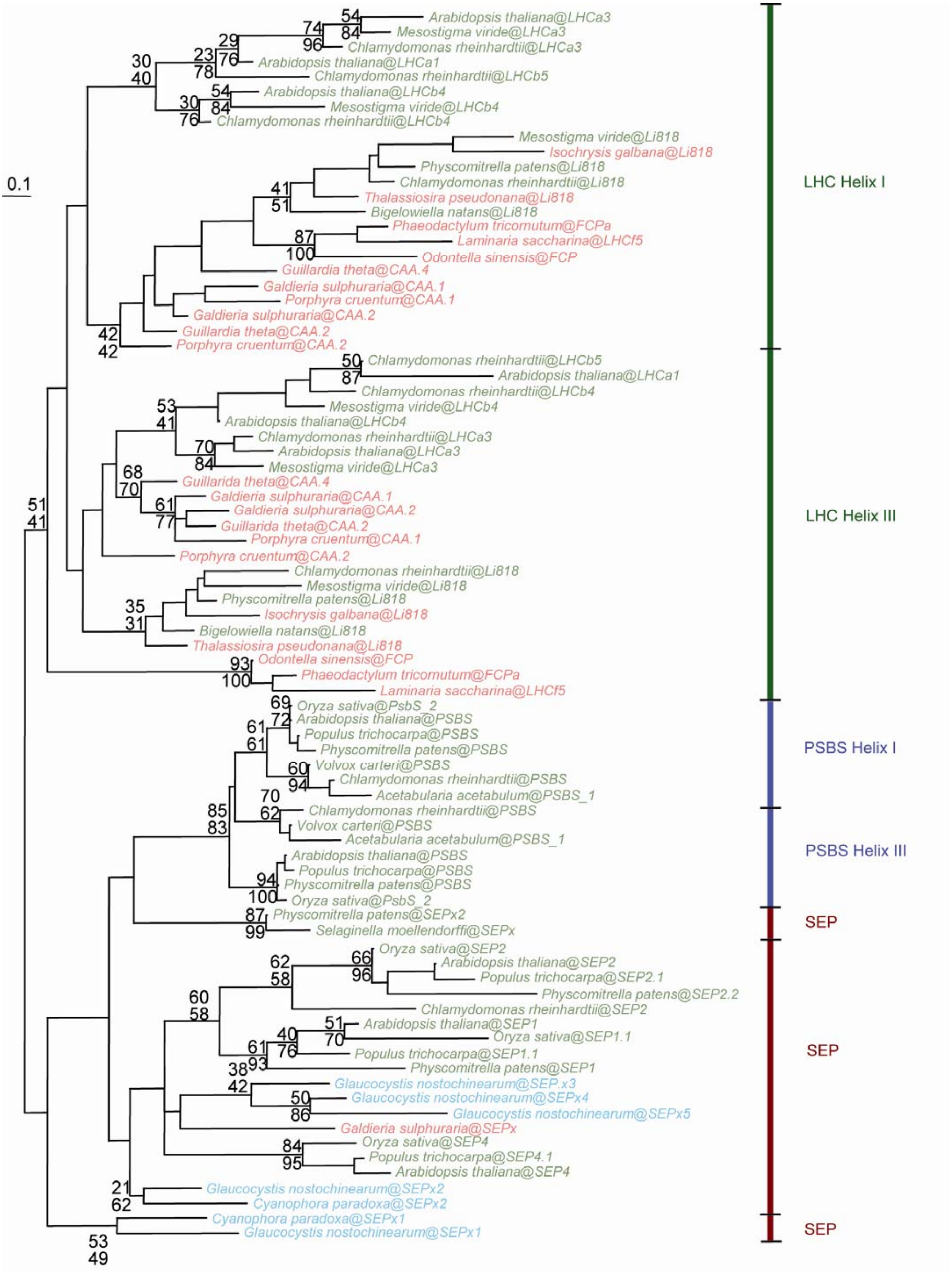

Figure S3

Phylogenetic analysis of the first and third helices of LHC and PSBS in the presence of representative SEP. A Maximum Likelihood Tree which corresponds to Figure 4B was inferred by PhyML using a

WAG+Γ4 model based on 82 sequences and 31 amino acid positions. Bootstrap values (100 replicates) were calculated both with (upper value) and without gamma (lower value) correction. For clarity, values below 40 and those of distal nodes were omitted. In a topology test this topology (above and Figure 4) was tested against the alternative topology shown in Supplemental Figure S2B in Additional data file 1 and was preferred with high significance (see main text).

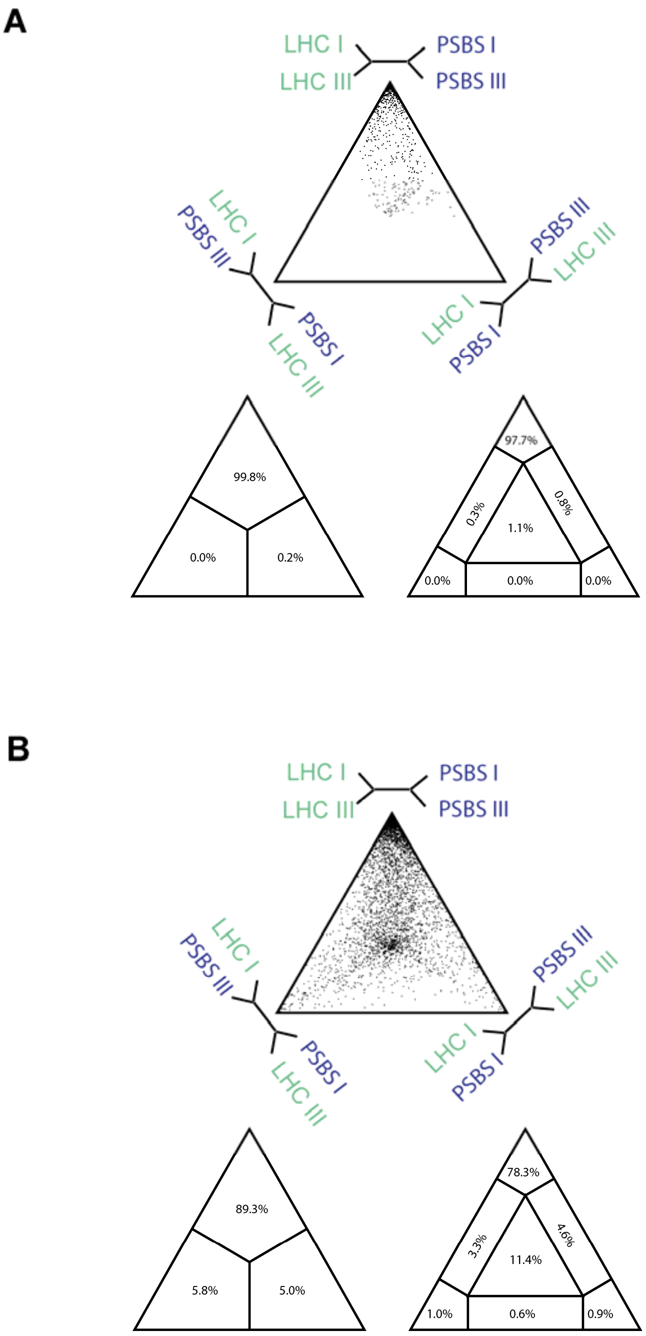

Figure S4

Additional four-cluster likelihood mapping analyses of individual CB motifs in LHC and PSBS similar to the analysis in Figure 4A. (A) Reduced dataset with the fast-evolving (long-branch) *Ostreococcus tauri* PSBS and the PSBS-like sequences from *V. carteri* and *C. reinhardtii,* as well as the divergent LHCz and FCP sequences removed. The percentage of unresolved quartets strongly decreased and the support for the clustering of helices I and III was improved. The topology with LHC helix I as sister group to LHC helix III is strongly supported with 99.8% over the one expected under a common origin with 0.2% in the three-partite diagram (or 97.7% over 0.0% in the seven-partite diagram). **(B)** Original dataset (120 CB helices) including fast-evolving sequences with three known functional amino acid positions (glutamate E+0, histidine/asparagines H/N+3 and arginine R+5) removed. The result is similar to the original analysis (Figure 4A) with the topology with LHC helix I as sister group to LHC helix III being strongly supported with 89.3% over the one expected under a common origin with 5.0% in the three-partite diagram (or 78,3% over 0.9% in the seven-partite diagram).

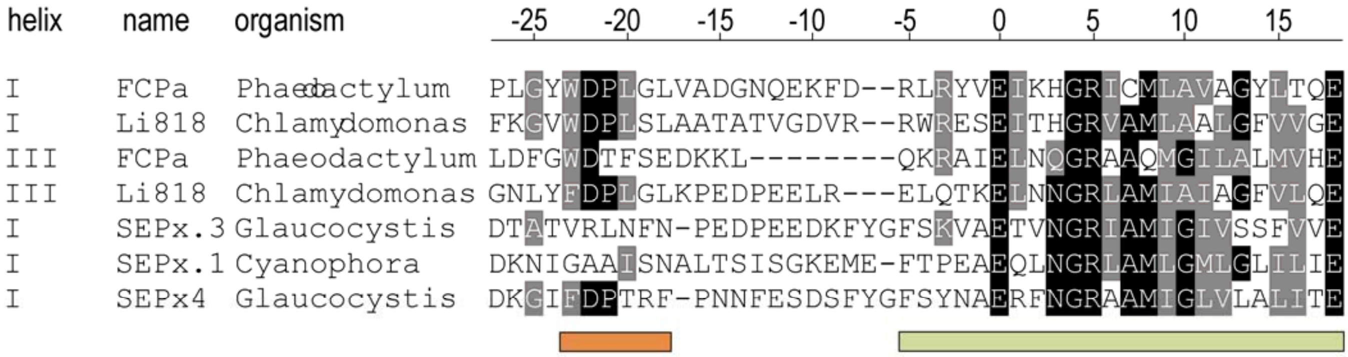

Figure S5

Alignment of LHC and SEP sequences. Conserved carotenoid-binding motifs (orange box, "FDPLGL" or similar) and CB motifs (green box) before first (I) and third (III) helices in two members of different LHC subfamilies, FCPa from the diatom *P. tricornutum* [accession Q08584] and Li818 from the green algae *C. reinhardtii* [accession Q03965]. Three typical SEPs from glaucophytes were added, one of them (SEPx.4) with a possible partial carotenoid-binding motif. Identical amino acids are surrounded by a black box, similar amino acids by a grey box. Note that this is only one of several (including stronger) indications that LHCs likely have evolved from SEPs.

Table S1: Non-LHC sequences of the extended LHC protein superfamily in 15 photosynthetic organisms

Note: Results for HMM and BlastP are given both as e-value and bit score.

Note on alternative names: The Lil names (light-harvesting like) for parts of the non-LHC proteins in Arabidopsis thaliana have been introduced by Jansson (1999) and extended by (Klimmek et al., 2006; Rensing et al., 2008; Alboresi et al., 2008) and others.
Corresponding names are: ELIP (Lil1), OHP1 (Lil2), SEP3 (Lil3), SEP1 (Lil4), SEP2 (Lil5), OHP2 (Lil6) and SEPx from Physcomitrella patens (Lil7).
The Lhl names for parts of the non-LHC sequences in Chlamydomonas rheinhardtii have been introduced by (Teramoto et al., 2004).
(Funk and Vermaas, 1999) used the name Scp (small CAB-like proteins) for the HLIPs in Synechocystis sp. PCC 6803.
For references see below.

*** Putative membrane anchor in OHP2**
**** Chlorophyll binding (CB) motifs additionally predicted as TM helix**
***** List of HMM profiles see below**
******For these ELIP or other sequences, additional highly similar copies may exist (e.g. tandem duplicates in Physcomitrella)**

organism with database	model	proposed name	alternative name	classified as	# TM helices	1st HMM***	2nd HMM	3rd HMM	1st BlastP	2nd BlastP	3rd BlastP	4th BlastP	comment on gene model	name in BlastP results
**Gloeobacter violaceus PCC 7421**	gsr2714	**HLIP1**		**HLIP**	1	**HLIPa**	SEP	LHL4	**Syn_HLIPD_**	Ana_HLIP3_	Glo_HLIP2_	Syn_HLIPC_	HLIP numbers newly assigned	Glo_HLIP1_
Cyanobase (Kazusa)						**3,40E-28**	1,60E-12	4,90E-10	**2,00E-12**	2,00E-12	3,00E-12	1,00E-09		
						**95,4**	43,4	35,1	**58,2**	58,2	57,8	48,9		
	gsl1443	**HLIP2**		**HLIP**	1	**HLIPa**	SEP	HLIPb	**Ana_HLIP3_**	Syn_HLIPC_	Syn_HLIPD_	Cme_HLIP__	HLIP numbers newly assigned	Glo_HLIP2_
						**2,30E-28**	2,20E-18	3,90E-13	**9,00E-14**	4,00E-13	6,00E-13	1,00E-12		
						**96**	62,8	45,4	**62,8**	60,5	60,1	59,3		
	gsl2056	**HLIP3**		**HLIP**	1	**HLIPb**	HLIPa	SEP	**Ana_HLIP5_**	Ana_HLIP6_	Ana_HLIP1_	Glo_HLIP6_	HLIP numbers newly assigned	Glo_HLIP3_
						**7,00E-41**	1,90E-14	4,60E-11	**2,00E-22**	2,00E-22	4,00E-21	4,00E-19		
						**137,6**	49,7	38,5	**91,7**	91,3	87	80,5		
	gsl0199	**HLIP4**		**HLIP**	1	**HLIPb**	HLIPa	RedCAP	**Glo_HLIP5_**	Ana_HLIP5_	Ana_HLIP6_	Ana_HLIP1_	HLIP numbers newly assigned; possible deletion in TM helix	Glo_HLIP4_
						**1,60E-15**	2,40E-07	0,00011	**7,00E-22**	2,00E-12	6,00E-12	4,00E-11		
						**53,3**	16,9	-9,4	**89,7**	58,2	56,6	53,9		
	gsl3261	**HLIP5**		**HLIP**	1	**HLIPb**	HLIPa	OHP2	**Glo_HLIP4_**	Ana_HLIP5_	Glo_HLIP3_	Ana_HLIP6_	HLIP numbers newly assigned; possible deletion in TM helix	Glo_HLIP5_
						**3,70E-16**	2,00E-06	0,00013	**7,00E-22**	6,00E-12	8,00E-12	2,00E-11		
						**55,4**	9,9	-12,2	**89,7**	56,6	56,2	55,1		
	gsr0742	**HLIP6**		**HLIP**	1	**HLIPb**	HLIPa	SEP	**Ana_HLIP6_**	Ana_HLIP5_	Ana_HLIP1_	Glo_HLIP3_	HLIP numbers newly assigned	Glo_HLIP6_
						**2,70E-40**	6,40E-13	6,10E-08	**3,00E-20**	1,00E-19	4,00E-19	4,00E-19		
						**135,6**	44,7	28,1	**84,3**	82	80,5	80,5		
	gll0839 (893846	**FerrochelataseII**		**FerrochelataseII**	2	**LHC**	SEP4	SEP5	**Syn_Ferro_**	Ana_Ferro_	Ppa_FerrII	Cre_FerroI	Ferrochelatases with correct stop codon but no CB motif	Glo_FerrII
	- 894829)					**0,87**	0,94	4,3	**5,00E-123**	8,00E-121	8,00E-113	1,00E-112	5´end partial	
						**-39,1**	-31,2	-33,2	**428**	421	394	394		
**Anabena sp. PCC 7120**	asl0873	**HLIP1**		**HLIP**	1	**HLIPb**	HLIPa	LHL4	**Ana_HLIP6_**	Ana_HLIP5_	Glo_HLIP3_	Glo_HLIP6_	HLIP named as in Lindell et al. (2004)	Ana_HLIP1_
Cyanobase (Kazusa)						**9,30E-41**	1,90E-11	6,30E-07	**1,00E-24**	1,00E-24	4,00E-21	4,00E-19		
						**137,1**	39,8	15,9	**99**	99	87	80,5		
	asl0514	**HLIP2**		**HLIP**	1	**HLIPa**	SEP	HLIPb	**Syn_HLIPB_**	Syn_HLIPA_	Glo_HLIP2_	Ana_HLIP4_	HLIP named as in Lindell et al. (2004)	Ana_HLIP2_
						**1,90E-26**	8,00E-14	6,70E-10	**6,00E-17**	1,00E-16	9,00E-11	8,00E-10		
						**89,6**	47,7	34,6	**73,2**	72	52,8	49,7		
	asl2354	**HLIP3**		**HLIP**	1	**HLIPa**	OHP1	SEP	**Syn_HLIPD_**	Glo_HLIP2_	Glo_HLIP1_	Gno_SEPx.5_	HLIP named as in Lindell et al. (2004)	Ana_HLIP3_
						**1,70E-28**	3,00E-14	2,80E-11	**3,00E-21**	9,00E-14	2,00E-12	1,00E-09		
						**96,5**	49,1	39,2	**87,4**	62,8	58,2	49,3		
	asr5262	**HLIP4**		**HLIP**	1	**HLIPb**	HLIPa	SEP	**Ana_HLIP7_**	Cpa_HLIP1_	Gsu_HLIP__	Ana_HLIP8_	HLIP named as in Lindell et al. (2004)	Ana_HLIP4_
						**4,10E-37**	3,00E-31	2,20E-14	**8,00E-18**	2,00E-16	7,00E-16	2,00E-15		
						**125,1**	105,6	49,5	**76,3**	71,6	69,7	68,2		
	asr3042	**HLIP5**		**HLIP**	1	**HLIPb**	HLIPa	LHL4	**Ana_HLIP6_**	Ana_HLIP1_	Glo_HLIP3_	Glo_HLIP6_	HLIP named as in Lindell et al. (2004)	Ana_HLIP5_
						**1,50E-40**	1,40E-12	2,40E-07	**7,00E-33**	1,00E-24	2,00E-22	1,00E-19		
						**136,5**	43,6	19	**126**	99	91,7	82		
	asr3043	**HLIP6**		**HLIP**	1	**HLIPb**	HLIPa	SEP	**Ana_HLIP5_**	Ana_HLIP1_	Glo_HLIP3_	Glo_HLIP6_	HLIP named as in Lindell et al. (2004)	Ana_HLIP6_
						**7,60E-41**	8,70E-13	7,40E-08	**7,00E-33**	1,00E-24	2,00E-22	3,00E-20		
						**137,4**	44,2	27,9	**126**	99	91,3	84,3		
	asl3726	**HLIP7**		**HLIP**	1	**HLIPa**	HLIPb	SEP	**Ana_HLIP4_**	Ana_HLIP8_	Syn_HLIPC_	Cpa_HLIP1_	HLIP named as in Lindell et al. (2004)	Ana_HLIP7_
						**7,50E-28**	1,70E-17	1,10E-11	**8,00E-18**	5,00E-15	4,00E-13	1,00E-12		
						**94,3**	59,8	40,6	**76,3**	67	60,5	58,9		
	asl0449	**HLIP8**		**HLIP**	1	**HLIPa**	HLIPb	SEP	**Ana_HLIP4_**	Ana_HLIP7_	Syn_HLIPC_	Cpa_HLIP1_	HLIP named as in Lindell et al. (2004)	Ana_HLIP8_
						**8,70E-22**	8,30E-11	1,70E-06	**2,00E-15**	5,00E-15	4,00E-11	1,00E-10		
						**74,1**	37,7	20,5	**68,2**	67	53,9	52,4		
	alr3751	**FerrochelataseII**		**FerrochelataseII**	2	**HLIPa**	HLIPb	FerroII	**Syn_Ferro_**	Ppa_FerrII	Ath_FerrII	Cre_FerroI	Ferrochelatase II with CB motif	Ana_Ferro_
						**2,20E-21**	4,50E-13	7,70E-13	**7,00E-172**	8,00E-133	6,00E-130	7,00E-129		
						**72,8**	45,2	44,4	**591**	461	451	448		
**Synechocystis sp. PCC 6803**	ssl2542	**HLIP A**	Scp-A	**HLIP**	1	**HLIPa**	SEP	SEPxMoss	**Syn_HLIPB_**	Ana_HLIP2_	Syn_HLIPD_	Glo_HLIP2_		Syn_HLIPA_
Cyanobase (Kazusa)						**2,00E-27**	1,60E-11	4,10E-09	**2,00E-34**	1,00E-16	3,00E-09	3,00E-09		
						**92,9**	40	32	**131**	72	47,8	47,8		
	ssr2595	**HLIP B**	Scp-B	**HLIP**	1	**HLIPa**	SEP	SEPxMoss	**Syn_HLIPA_**	Ana_HLIP2_	Syn_HLIPD_	Ana_HLIP3_		Syn_HLIPB_
						**3,50E-27**	1,00E-10	5,00E-08	**2,00E-34**	7,00E-17	5,00E-09	9,00E-09		
						**92,1**	37,3	28,4	**131**	73,2	47	46,2		
	ssl1633	**HLIP C**	Scp-C	**HLIP**	1	**HLIPa**	SEP	LHL4	**Gsu_HLIP__**	Cpa_HLIP1_	Ana_HLIP4_	Ana_HLIP7_		Syn_HLIPC_
						**6,30E-29**	5,80E-12	1,20E-10	**4,00E-14**	2,00E-13	3,00E-13	4,00E-13		
						**97,9**	41,5	37,1	**63,9**	61,6	60,8	60,5		
	ssr1789	**HLIP D**	Scp-D	**HLIP**	1	**HLIPa**	SEP	SEPxGlauco	**Ana_HLIP3_**	Glo_HLIP2_	Glo_HLIP1_	Gno_SEPx.5_		Syn_HLIPD_
						**7,00E-29**	1,80E-11	2,30E-09	**3,00E-21**	6,00E-13	2,00E-12	2,00E-09		
						**97,7**	39,9	32,9	**87,4**	60,1	58,2	48,5		
	slr0839	**FerrochelataseII**		**FerrochelataseII**	2	**HLIPa**	HLIPb	FerroII	**Ana_Ferro_**	Ath_FerrII	Cre_FerroI	Ppa_FerrII	Ferrochelatase II with CB motif	Syn_Ferro_
						**2,00E-20**	1,80E-14	1,20E-10	**7,00E-172**	2,00E-128	1,00E-127	2,00E-126		
						**69,6**	49,9	37,1	**591**	446	444	440		
**Cyanophora paradoxa**	P48367 (Uniprot)	**HLIP**	ycf17	**HLIP**	1	**HLIPa**	HLIPb	SEP	**Gsu_HLIP__**	Ana_HLIP4_	Syn_Ferro_	Cme_HLIP__	complete CDS	Cpa_HLIP1_
NCBI TPA						**2,70E-28**	2,40E-14	1,40E-11	**6,00E-20**	2,00E-16	4,00E-14	9,00E-14		
						**95,8**	49,4	40,3	**83,2**	71,6	63,9	62,8		
	BK006744	**OHP1-like1**		**OHP1-like**	1	**HLIPa**	SEP	SEPxGlauco	**Cpa_OHP1-like2**	Gno_SEPx3_	Gno_SEPx6_	Ana_HLIP4_	5' end partial but TP predicted	Cpa_OHP1-like1
						**3,00E-15**	1,80E-10	1,00E-08	**1,00E-49**	2,00E-09	6,00E-08	1,00E-07		
						**52,4**	36,5	30,7	**183**	49,7	45,1	44,3		
	BK006745	**OHP1-like2**		**OHP1-like**	1	**HLIPa**	SEP	SEPxGlauco	**Cpa_OHP1-like1**	Syn_HLIPC_	Ath_FerrII	Ppa_FerrII	5' end partial but TP predicted	Cpa_OHP1-like2
						**2,90E-16**	2,00E-11	3,80E-08	**2,00E-49**	8,00E-08	1,00E-07	1,00E-07		
						**55,8**	39,7	28,8	**183**	44,7	44,3	44,3		
	BK006746	**OHP2**	Lil6	**OHP2**	1	**OHP2**	SEP	HLIPa	**Gsu_OHP2__**	Tps_OHP2__	Pta_OHP2__	Olu_OHP2__	complete CDS	Cpa_OHP2__

						**9,80E-35**	2,00E-09	8,40E-08	**8,00E-12**	1,00E-09	7,00E-09	9,00E-09		
						**117,1**	33,1	20,3	**58,5**	51,6	48,9	48,5		
	BK006747	**SEPx.1**		**SEP**	**2**	**SEP**	HLIPa	HLIPb	**Gno_SEPx.1_**	Glo_HLIP2_	Cpa_OHP1-like1	Ana_HLIP4_	complete CDS, additional highly similar gene copies (e.g. ES236587.1) may exist	Cpa_SEPx1_
						**1,60E-18**	2,80E-13	4,70E-07	**2,00E-20**	2,00E-08	6,00E-07	6,00E-07		
						**63,3**	45,8	23,9	**87**	46,6	42	42		
	BK006748	**SEPx.2**		**SEP**	**2**	**SEPxGlauco**	SEP	HLIPa	**Gno_SEPx.2_**	Gno_SEPx.6_	Gno_SEPx.5_	Gno_SEPx.3_	complete CDS; additional highly similar gene copies (e.g. ES234255.1) may exist	Cpa_SEPx.2_
						**2,00E-36**	5,50E-24	8,90E-12	**1,00E-24**	2,00E-15	6,00E-12	1,00E-11		
						**122,7**	81,4	40,9	**100**	70,1	58,5	57,4		
	from EST EC660148 +	**FerrochelataseII**		**FerrochelataseII**	**2**	**HLIPa**	OHP1	OHP2	**Ana_Ferro_**	Syn_Ferro_	Osa_FerrII	Ath_FerrII	Ferrochelatase II with CB motif	Cpa_FerrII
						**5,90E-10**	8,80E-06	1,60E-05	**9,00E-102**	4,00E-100	2,00E-92	2,00E-92		
						**34,8**	10,5	-5,1	**357**	352	327	327		
**Glaucocystis nostochinearum**	BK006749	**SEPx.1**		**SEP**	**2**	**SEP**	HLIPa	FerroII	**Cpa_SEPx.1_**	Osa_FerrII	Ath_FerrII	Ppa_FerrII	complete CDS	Gno_SEPx.1_
NCBI TPA						**1,20E-19**	6,80E-13	1,50E-07	**2,00E-20**	1,00E-08	1,00E-08	1,00E-08		
						**67**	44,6	24,2	**87**	47,8	47,8	47,8		
	BK006750	**SEPx.2**		**SEP**	**2**	**SEPxGlauco**	SEP	HLIPa	**Cpa_SEPx.2_**	Gno_SEPx.6_	Gno_SEPx.5_	Gno_SEPx.4_	missing stop codon, otherwise complete	Gno_SEPx.2_
						**1,00E-36**	1,30E-24	4,30E-13	**1,00E-24**	3,00E-17	2,00E-11	6,00E-11		
						**123,7**	83,5	45,3	**100**	76,3	57	55,1		
	BK006751	**SEPx.3**		**SEP**	**2**	**SEPxGlauco**	SEP	ELIP	**Gno_SEPx4_**	Cpa_SEPx2_	Cpa_OHP1-like1	Ath_PSBS__	complete CDS	Gno_SEPx.3_
						**4,40E-37**	2,10E-21	3,70E-15	**3,00E-13**	1,00E-11	3,00E-09	5,00E-09		
						**125**	72,9	52,1	**63,2**	57,4	49,7	48,9		
	BK006752	**SEPx.4**		**SEP**	**2**	**SEPxGlauco**	SEP	HLIPa	**Gno_SEPx.3_**	Gno_SEPx.6_	Cpa_SEPx.2_	Gno_SEPx.2_	5' end partial; otherwise complete	Gno_SEPx.4_
						**1,70E-38**	1,40E-22	1,40E-12	**1,00E-13**	2,00E-11	3,00E-11	4,00E-11		
						**129,6**	76,7	43,6	**63,2**	56,2	55,5	55,1		
	BK006753	**SEPx.5**		**SEP**	**2**	**SEPxGlauco**	SEP	OHP2	**Gno_SEPx.6_**	Cpa_SEPx.2_	Gno_SEPx.2_	Gno_SEPx.4_	complete CDS	Gno_SEPx.5_
						**1,10E-35**	4,40E-23	4,50E-15	**4,00E-20**	6,00E-12	2,00E-11	4,00E-10		
						**120,3**	78,4	51,8	**85,5**	58,5	57	52,4		
	BK006754	**SEPx.6**		**SEP**	**2**	**SEPxGlauco**	SEP	ELIP	**Gno_SEPx.5_**	Gno_SEPx.2_	Cpa_SEPx.2_	Gno_SEPx.4_	complete CDS	Gno_SEPx.6_
						**4,00E-35**	7,50E-22	2,50E-11	**5,00E-20**	3,00E-17	2,00E-15	3,00E-11		
						**118,5**	74,4	39,4	**85,5**	76,3	70,1	56,2		
	from EST EC118146	**FerrochelataseII**		**FerrochelataseII**	**1**	**HLIPa**	HLIPb	FerroII	**Cpa_FerrII**	Cre_FerroI	Ana_Ferro_	Syn_Ferro_	5' end partial; Ferrochelatase II with CB motif	Gno_FerrII
						**3,30E-07**	4,00E-05	4,50E-05	**4,00E-26**	2,00E-12	3,00E-12	8,00E-11		
						**15,8**	9,1	2,5	**104**	58,5	58,2	53,5		
**Galdieria sulphuraria**	stig_35:42202..42401	**HLIP**	ycf17	**HLIP**	**1**	**HLIPa**	HLIPb	SEP	**Cpa_HLIP1_**	Ana_HLIP4_	Syn_HLIPC_	Syn_Ferro_	complete CDS; plastid located.	Gsu_HLIP__
Built 3 Aug 2007						**1,10E-31**	4,30E-17	1,30E-12	**6,00E-20**	7,00E-16	4,00E-14	5,00E-14		
http://genomics.msu.edu/galdieria/						**107**	58,5	43,6	**83,2**	69,7	63,9	63,5		
	Gs06110.2	**OHP2**	Lil6	**OHP2**	**1 (2*)**	**OHP2**	SEPxGlauco	HLIPa	**Cme_OHP2__**	Ptr_OHP2__	Tps_OHP2__	Cpa_OHP2__	complete CDS; different variants annotated.	Gsu_OHP2__
						**1,10E-38**	1,80E-12	3,20E-11	**2,00E-17**	9,00E-17	4,00E-15	6,00E-12		
						**130,3**	43,2	39	**76,6**	74,7	69,3	58,5		
	Gs28440.1	**SEPx**		**SEP**	**2**	**SEP**	ELIP	HLIPa	**Gno_SEPx.5_**	Gno_SEPx.6_	Cpa_SEPx.2_	Gno_SEPx.2_	TP and twin arginine signal unclear.	Gsu_SEPx__
						**2,20E-21**	5,00E-09	1,20E-08	**5,00E-09**	2,00E-07	3,00E-07	3,00E-07		
						**72,8**	31,7	26,8	**48,1**	43,1	42,4	42		
	Gs25280.1	**OHP1-like**		**OHP1-like**	**1**	**HLIPa**	SEP	LHL4	**Ana_HLIP2_**	Glo_HLIP2_	Ana_HLIP4_	Glo_HLIP1_	complete CDS, TP and twin arginine present; nuclear-encoded OHP1-like	Gsu_OHP1-like
						**6,70E-11**	5,00E-09	1,60E-07	**3,00E-08**	9,00E-07	3,00E-06	6,00E-06		
						**38**	31,7	20,2	**45,8**	40,8	38,9	38,1		
	Gs47790.1	**RedCAP**		**RedCAP**	**3**	**RedCAP**	HLIPa	HLIPb	**Tps_RedCAP_**	Ptr_RedCAP___	Ppa_PSBS__	Osa_PsbS,2	complete CDS; predicted three-helix CB protein	Gsu_RedCAP__G
						**3,60E-46**	2,00E-15	2,90E-11	**3,00E-46**	3,00E-42	2,00E-09	4,00E-08		
						**155,1**	53	39,2	**172**	159	50,8	46,2		
	Gs58560.1	**Ferrochelatase II**		**Ferrochelatase II**	**1**	**SEPxMoss**	LHC	ELIP	**Cme_FerrII**	Ath_FerrII	Syn_Ferro_	Cre_FerroI	FerrochelataseII with correct stop codon but apparently no CB motif	Gsu_FerrII
						**1,2**	1,6	3,2	**9,00E-126**	7,00E-40	3,00E-39	2,00E-38		
						**-17,8**	-41,2	-36,9	**438**	152	150	147		
**Cyanidioschyzon merolae**	CMV110C	**HLIP**	ycf17	**HLIP**	**1**	**HLIPa**	SEP	SEPxGlauco	**Cpa_HLIP1_**	Gsu_HLIP__	Glo_HLIP2_	Syn_HLIPC_	complete CDS	Cme_HLIP__
http://merolae.biol.s.u-tokyo.ac.jp						**3,80E-23**	6,20E-12	5,80E-09	**9,00E-14**	6,00E-13	1,00E-12	2,00E-10		
						**78,7**	41,4	31,5	**62,8**	60,1	59,3	52		
	CMP191C	**OHP2**	Lil6	**OHP2**	**1 (2*)**	**OHP2**	SEP	SEPxGlauco	**Ptr_OHP2__**	Tps_OHP2__	Gsu_OHP2__	Ppa_OHP2__	complete CDS with twin arginine motif and TP	Cme_OHP2__
						**1,10E-38**	4,40E-10	9,20E-10	**5,00E-21**	4,00E-20	3,00E-17	1,00E-14		
						**130,3**	35,2	34,2	**89**	85,9	76,6	67,8		
	CMS035C	**Ferrochelatase II**		**FerrochelataseII**	**1**	**SEPxMoss**	SEP	SEP3	**Gsu_FerrII**	Syn_Ferro_	Ath_FerrII	Ppa_FerrII	correct stop codon but apparently no CB motif; complete CDS with twin	Cme_FerrII
						**2**	2,1	2,8	**8,00E-126**	1,00E-43	6,00E-36	4,00E-35	arginine motif and TP	
						**-19,6**	-32,9	-18,2	**438**	165	139	137		
**Phaeodactylum tricornutum**	Phatr2:53712	**OHP1-like1**		**OHP1-like**	**1**	**ELIP**	SEP	SEPxGlauco	**Gno_SEPx.3_**	Cre_ELIP6_	Tps_OHP1-like	Ath_PSBS__	nuclear-encoded OHP1-like , long-branching	Ptr_OHP1-like1
JGI Phaeo2						**1,30E-06**	3,00E-06	4,60E-06	**1,00E-05**	7,00E-05	7,00E-05	3,00E-04		
						**18,1**	18,4	9,4	**37**	34,3	34,3	32,3		
	Phatr2:33932	**OHP1-like2**		**OHP1-like**	**1**	**FerroII**	SEP	ELIP	**Tps_OHP1-like**	Ath_SEP3,2	Ath_SEP3,1	Ptr_OHP1-like1	nuclear-encoded OHP1-like, long-branching	Ptr_OHP1-like2
						**0,00019**	0,0002	0,00069	**7,00E-09**	0,004	0,004	0,005		
						**-3,1**	2,4	-5,2	**48,5**	29,3	29,3	28,9		
	Phatr2:55112	**OHP2**	Lil6	**OHP2**	**1 (2*)**	**OHP2**	SEP	SEPxGlauco	**Tps_OHP2__**	Cme_OHP2__	Gsu_OHP2__	Osa_OHP2__		Ptr_OHP2__
						**4,70E-38**	2,60E-10	2,70E-10	**4,00E-46**	4,00E-21	8,00E-17	5,00E-09		
						**128,2**	36	36	**172**	89	74,7	48,9		
	Phatr2:56446	**SEPx**		**SEP**	**2**	**HLIPa**	SEPxGlauco	RieskeCB	**Tps_SEP___**	Gsu_HLIP__	Cre_PsbSlk	Ana_Ferro_		Ptr_SEP___
						**8,90E-08**	2,00E-06	3,10E-06	**5,00E-30**	4,00E-07	7,00E-07	9,00E-07		
						**20,1**	12,8	18,6	**118**	42	41,2	40,8		
	Phatr2:17326	**RedCAP**		**RedCAP**	**3**	**RedCAP**	HLIPa	HLIPb	**Tps_RedCAP_**	Gsu_RedCAP__G	Osa_ELIP2_	Ath_FerrII	predicted three-helix protein	Ptr_RedCAP___
						**1,40E-47**	4,70E-11	4,20E-07	**1,00E-72**	3,00E-42	2,00E-05	2,00E-05		
						**159,9**	38,5	24,2	**260**	159	37,7	37,4		
	Phatr2:45357	**FerrochelataseII**		**FerrochelataseII**	**2**	**SEP**	SEPxGlauco	ELIP	**Tps_FerrII**	Ath_FerrII	Cre_FerroI	Osa_FerrII	CB motif rather divergent	Ptr_FerrII
						**5,30E-05**	6,50E-05	6,50E-05	**7,00E-132**	4,00E-112	1,00E-111	2,00E-110		
						**7,5**	-1,3	3,5	**458**	393	391	387		
**Thalassiosira pseudonana**	Thaps3:270237	**OHP1-like**		**OHP1-like**	**1**	**SEP**	SEP12	LHL4	**Ptr_OHP1-like2**	Ptr_OHP1-like1	Osa_FerrII	Vca_LHL4__	nuclear-encoded OHP1-like, long-branching	Tps_OHP1-like
JGI Thapsi3						**9,40E-06**	4,00E-05	6,20E-05	**7,00E-09**	1,00E-04	3,00E-04	7,00E-04		
						**14**	1,7	1,5	**48,5**	34,3	33,1	32		
	Thaps3:270213	**OHP2**	Lil6	**OHP2**	**1**	**OHP2**	SEPxGlauco	SEP	**Ptr_OHP2__**	Cme_OHP2__	Gsu_OHP2__	Cpa_OHP2__		Tps_OHP2__
						**2,20E-39**	1,40E-07	5,20E-07	**4,00E-46**	4,00E-20	4,00E-15	8,00E-10		
						**132,6**	23,7	25	**172**	85,9	69,3	51,6		
	Thaps3:270212	**SEPx**		**SEP**	**2**	**SEP**	HLIPa	SEPxGlauco	**Ptr_SEP___**	Cpa_SEPx2_	Ana_Ferro_	Gsu_HLIP__		Tps_SEP___
						**8,90E-09**	1,80E-08	2,00E-07	**5,00E-30**	7,00E-08	1,00E-07	2,00E-07		
						**30,9**	25,5	22,1	**118**	44,7	43,9	43,5		
	Thaps3:270215	**RedCAP**		**RedCAP**	**3**	**RedCAP**	HLIPb	HLIPa	**Ptr_RedCAP_**	Gsu_RedCAP__G	Ana_HLIP4_	Tps_SEP___	predicted three-helix protein	Tps_RedCAP___
						**1,60E-47**	2,30E-09	4,00E-08	**1,00E-72**	3,00E-46	7,00E-06	9,00E-06		
						**159,6**	32,9	22,8	**260**	172	38,9	38,5		
	Thaps3:21006	**FerrochelataseII**		**FerrochelataseII**	**2**	**SEP**	HLIPa	SEP12	**Ptr_FerrII**	Ppa_FerrII	Ath_FerrII	Osa_FerrII	Ferrochelatase II with CB motif	Tps_FerrII
						**2,70E-08**	1,80E-07	7,40E-06	**5,00E-132**	6,00E-84	2,00E-80	7,00E-80		
						**29,3**	17,9	7,4	**458**	298	287	285		
**Chlamydomonas reinhardtii**	Chlre4:133963	**OHP1**	Lil2, Lhl2	**OHP1**	**1**	**OHP1**	HLIPa	SEP	**Olu_OHP1__**	Ppa_OHP1,2	Osa_OHP1,2	Ath_OHP1__	complete CDS	Cre_OHP1__
JGI Chlre 4.0						**9,80E-36**	1,50E-15	2,30E-10	**1,00E-16**	6,00E-16	1,00E-14	3,00E-14		
						**120,5**	53,4	36,2	**73,2**	70,9	66,2	65,5		
	Chlre4:187308	**OHP2**	Lil6	**OHP2**	**1**	**OHP2**	SEP	HLIPa	**Ppa_OHP2__**	Pta_OHP2__	Olu_OHP2__	Ath_OHP2__	5´end partial	Cre_OHP2__
						**7,30E-28**	1,60E-07	6,60E-06	**2,00E-22**	9,00E-22	8,00E-21	9,00E-19		
						**94,3**	26,8	5,9	**91,3**	89,4	86,3	79,3		
	Chlre4:184228	**SEP2**	Lil5	**SEP**	**2**	**SEP12**	SEP	SEPxMoss	**Ppa_SEP2,1**	Ppa_SEP2,2	Osa_SEP2__	Pta_SEP2__	complete CDS	Cre_SEP2__
						**4,90E-27**	5,30E-18	1,90E-06	**1,00E-10**	2,00E-09	7,00E-07	9,00E-07		
						**91,6**	61,6	23,2	**54,7**	50,8	42	41,6		
	Chlre4:185309	**SEP3**	Lil3, Lhl3	**SEP**	**2**	**SEP3**	SEP	HLIPa	**Ppa_SEP3,2**	Ppa_SEP3,3	Ppa_SEP3,1	Osa_SEP3__	complete CDS	Cre_SEP3__
						**4,80E-37**	2,00E-24	1,00E-07	**3,00E-59**	3,00E-58	1,00E-55	3,00E-53		
						**124,8**	82,9	19,7	**216**	213	204	196		
	Chlre4:183986	**ELIP1**	Lil1, Lhl1	**ELIP**	**3****	**ELIP**	HLIPa	SEP	**Cre_ELIP2_**	Pta_ELIP2_	Cre_ELIP6_	Osa_ELIP1_	complete CDS; ELIP number newly assigned	Cre_ELIP1_
						**1,00E-25**	3,40E-08	2,70E-06	**3,00E-14**	6,00E-14	5,00E-13	4,00E-13		

						87,2	23,3	18,8	66,6	65,5	62,4	62,8		
	Chlre4:191585	ELIP2	Lil1, Lhl1	ELIP	3**	ELIP	SEP	OHP2	Cre_ELIP6_	Osa_ELIP3_	Osa_ELIP1_	Osa_ELIP2_	complete CDS; ELIP number newly assigned	Cre_ELIP2_
						1,90E-26	4,70E-06	9,90E-06	6,00E-18	6,00E-17	1,00E-16	4,00E-16		
						89,6	16,7	-3,4	78,6	75,1	74,3	72,4		
	Chlre4:191226	ELIP3	Lil1, Lhl1	ELIP	3	ELIP	HLIPa	SEP	Cre_ELIP5_	Olu_ELIP7	Cre_ELIP7_	Olu_ELIP2_	complete CDS; ELIP number newly assigned	Cre_ELIP3_
						4,10E-26	6,50E-07	9,20E-07	2,00E-40	4,00E-15	4,00E-11	5,00E-09		
						88,5	13,6	22,9	154	70,1	56,6	49,7		
	Chlre4:392377	ELIP4	Lil1, Lhl1	ELIP	3	ELIP	SEP	HLIPa	Osa_ELIP1_	Ath_ELIP1_	Osa_ELIP2_	Pta_ELIP1_	ELIP number newly assigned	Cre_ELIP4_
						8,60E-25	3,70E-16	1,40E-07	6,00E-19	9,00E-17	2,00E-16	8,00E-16		
						84,1	55,4	18,7	82	74,7	73,6	71,6		
	Chlre4:151801	ELIP5	Lil1, Lhl1	ELIP	3	ELIP	HLIPa	SEP	Cre_ELIP3_	Olu_ELIP7	Cre_ELIP1_	Osa_ELIP3_	ELIP number newly assigned	Cre_ELIP5_
						1,20E-24	1,80E-08	1,90E-07	2,00E-40	4,00E-21	1,00E-12	3,00E-11		
						83,6	25,4	26,5	154	89,7	61,2	57		
	Chlre4:182724	ELIP6	Lil1, Lhl1	ELIP	3	ELIP	SEP	HLIPa	Cre_ELIP2_	Pta_ELIP2_	Cre_ELIP4_	Osa_ELIP1_	complete CDS; ELIP number newly assigned	Cre_ELIP6_
						5,30E-23	1,20E-10	4,40E-06	5,00E-18	3,00E-16	2,00E-15	9,00E-15		
						78,2	37,1	7,3	78,6	72,8	70,1	67,8		
	Chlre4:148418	ELIP7	Lil1, Lhl1	ELIP	3	ELIP	HLIPa	SEP	Ath_ELIP2_	Ath_ELIP1_	Pta_ELIP1_	Pta_ELIP2_	ELIP number newly assigned; 5´end partial	Cre_ELIP7_
						2,70E-14	2,80E-08	3,70E-08	3,00E-18	6,00E-18	1,00E-15	2,00E-15		
						49,3	23,9	28,9	78,6	77,8	70,1	69,7		
	Chlre4:137786	ELIP8	Lil1, Lhl1	ELIP	3	ELIP	HLIPa	SEP	Ath_ELIP1_	Pta_ELIP1_	Pta_ELIP2_	Osa_ELIP3_	complete CDS; ELIP number newly assigned;	Cre_ELIP8_
						1,10E-28	7,20E-07	1,20E-05	2,00E-20	2,00E-18	1,00E-17	2,00E-17		
						97	13,2	13,1	87	80,1	77,4	77		
	Chlre4:148916	ELIP9	Lil1, Lhl1	ELIP	3	ELIP	HLIPa	SEP	Olu_ELIP1_	Pta_ELIP2_	Osa_ELIP1_	Ath_ELIP1_	complete CDS; ELIP number newly assigned	
						1,40E-24	6,2E-22	3,50E-20	2,00E-12	3,00E-11	8,00E-11	1,00E-10		
						83,4	74,6	68,8	47	49	49	50		
	Chlre4:139895	Lhl4		Lhl4	3	LHL4	SEP	HLIPa	Vca_LHL4__	Ppa_SEPx.1_	Gno_SEPx.2_	Cpa_SEPx.2_	complete CDS	Cre_LHL4__
						5,80E-45	6,60E-26	2,20E-23	1,00E-101	8,00E-10	8,00E-10	3,00E-09		
						151,1	87,8	79,5	357	52,4	52,4	50,4		
	Chlre4:196341	PSBS1****	CP22	PSBS	4**	PSBS	SEP	SEPxMoss	Ppa_PSBS__	Ath_PSBS__	Osa_PsbS,1	Osa_PsbS,1	Additional nearly identical gene copy PSBS2: Chlre4:171516	Cre_PSBS1__
						1,50E-44	4,60E-17	3,10E-15	2,00E-43	9,00E-43	1,00E-41	6,00E-18		
						149,7	58,4	52,4	162	160	157	78,6		
	Chlre4:175221	PSBS-like		PSBS	4	PSBS	SEP	LHL4	Vca_PSBSlk	Cre_PSBS1__	Pta_PSBS2_	Ath_PSBS__	complete CDS; rather divergent PSBS with close homolog in Volvox carteri	Cre_PSBSlk
						3,40E-12	5,00E-09	1,30E-07	1,00E-105	9,00E-26	6,00E-16	1,00E-15		
						42,3	31,7	21	370	105	72,8	72		
	Chlre4:192099	Rieske-like		Rieske-like	2	SEP4	ELIP	RedCAP	Olu_Rieske	Ath_Rieske	Osa_Rieske	Ppa_Rieske	no CB motif, complete CDS	Cre_Rieske
						0,072	0,45	0,83	4,00E-54	2,00E-38	2,00E-37	5,00E-37		
						-20,8	-29,3	-44,8	199	147	144	142		
	Chlre4:183446	FerrochelataseII		FerrochelataseII	2	FerroII	HLIPa	SEP	Ppa_FerrII	Ath_FerrII	Osa_FerrII	Olu_FerrII	FerrochelataseII with CB motif; 5 end unclear	Cre_Ferrol
						4,90E-47	2,50E-16	2,70E-09	1,00E-169	3,00E-164	2,00E-163	5,00E-141		
						158	56	32,6	584	566	563	489		
**Ostreococcus lucimarinus**	Ost_Chr_	OHP1	Lil2	OHP1	1	OHP1	SEP	HLIPa	Cre_OHP1__	Ath_OHP1__	Ppa_OHP1,1	Ppa_OHP1,2		Olu_OHP1__
JGI Ost 2.0	_6|112051|112434					4,70E-19	3,10E-10	3,40E-10	2,00E-16	4,00E-13	8,00E-13	2,00E-12		
						65,1	35,8	35,6	73,2	62	60,8	59,3		
	Ost9901_3:9364	OHP2	Lil6	OHP2	1	OHP2	LHL4	HLIPa	Ppa_OHP2__	Pta_OHP2__	Ath_OHP2__	Cre_OHP2__		Olu_OHP2__
						2,70E-39	2,20E-07	7,00E-07	3,00E-24	1,00E-23	2,00E-21	8,00E-21		
						132,3	19,2	13,4	97,8	95,9	88,6	86,3		
	Ost9901_3:33858	SEP3	Lil3	SEP	2	SEP3	SEP	HLIPa	Cre_SEP3__	Ppa_SEP3,3	Ppa_SEP3,1	Ppa_SEP3,2		Olu_SEP3__
						7,20E-36	1,10E-20	1,90E-06	5,00E-53	1,00E-51	5,00E-51	8,00E-49		
						120,9	70,5	10	194	190	188	181		
	Ost9901_3:37301	ELIP1	Lil1	ELIP	3	ELIP	HLIPa	SEP	Cre_ELIP7_	Pta_ELIP2_	Pta_ELIP1_	Cre_ELIP6_	5´end partial; ELIP number newly assigned	Olu_ELIP1_
						3,30E-20	3,70E-09	8,30E-08	5,00E-13	1,00E-12	4,00E-12	4,00E-12		
						68,9	30,6	27,7	61,6	60,1	58,5	58,5		
	Ost9901_3:29720	ELIP2****	Lil1	ELIP	3**	SEP	ELIP	HLIPa	Ath_ELIP1_	Pta_ELIP1_	Osa_ELIP1_	Osa_ELIP2_	highly similar copies on chr. 13 and chr. 21; ELIP number newly assigned	Olu_ELIP2_
						5,60E-13	3,40E-11	4,50E-08	7,00E-13	7,00E-13	1,00E-11	2,00E-11		
						44,9	39	22,4	62	62	58,2	57,4		
	Ost9901_3:29644	ELIP3****	Lil1	ELIP	3**	ELIP	SEPxGlauco	OHP2	Cre_ELIP7_	Osa_ELIP2_	Cre_ELIP2_	Osa_ELIP3_	5´end partial; highly similar copies on chr. 13 and chr. 21; ELIP number newly assigned	Olu_ELIP3_
						1,60E-25	7,10E-06	9,50E-06	1,00E-14	3,00E-13	4,00E-13	5,00E-13		
						86,5	7,7	-3,3	67,4	62,4	62	61,6		
	Ost9901_3:92730	ELIP4	Lil1	ELIP	3	ELIP	OHP2	SEP	Pta_ELIP1_	Olu_ELIP1_	Pta_ELIP2_	Cre_ELIP7_	erroneously annotated as fusion at JGI; first ELIP CB motif rather divergent	Olu_ELIP4_
						1,20E-11	3,90E-07	8,40E-07	8,00E-10	4,00E-09	5,00E-09	1,00E-08		
						40,4	7,7	23,2	53,5	51,2	50,8	49,3		
	Ost9901_3:94034	ELIP5	Lil1	ELIP	3**	HLIPa	SEP	LHC	Olu_ELIP1_	Olu_ELIP3_	Olu_ELIP2_	Cre_ELIP5_	5'end partial; two CB motifs, but the first one very divergent; ELIP number newly assigned	Olu_ELIP5
						1,50E-05	3,80E-05	0,00011	4,00E-10	8,00E-08	1,00E-07	2,00E-07		
						9	0,2	4,8	52	44,3	43,9	43,1		
	Ost9901_3:24978	ELIP6	Lil1	ELIP	3	ELIP	SEPxGlauco	OHP2	Cre_ELIP1_	Cre_ELIP5_	Olu_ELIP7	Cre_ELIP3_	rather divergent ELIP; ELIP number newly assigned	Olu_ELIP6
						1,10E-06	3,50E-05	4,00E-05	1,00E-10	1,00E-06	1,00E-06	1,00E-06		
						11,7	5,9	0,7	54,7	41,6	41,2	41,2		
	Ost9901_3:26687	ELIP7	Lil1	ELIP	3	HLIPa	SEP	SEP3	Cre_ELIP5_	Cre_ELIP3_	Cre_ELIP7_	Cre_ELIP1_	; ELIP number newly assigned	Olu_ELIP7
						3,60E-07	6,90E-05	0,00012	3,00E-21	2,00E-15	7,00E-10	4,00E-09		
						22,9	-1,8	4,3	89,7	70,1	52	49,3		
	Ost9901_3:15953	PSBS	CP22	PSBS	4**	SEP	PSBS	SEP12	Cre_PSBS1__	Ath_PSBS__	Osa_PsbS,1	Pta_PSBS2_		Olu_PSBS__
						1,20E-06	1,70E-06	3,60E-05	3,00E-11	5,00E-11	9,00E-11	1,00E-10		
						21,8	15,4	2,2	56,6	55,8	55,1	54,7		
	Ost9901_3:86880	Rieske-like		Rieske-like	2	SEPxGlauco	LHL4	SEP	Cre_Rieske	Osa_Rieske	Ath_Rieske	Ppa_Rieske	no CB motif, complete CDS	Olu_Rieske
						0,47	0,83	1,2	3,00E-54	4,00E-38	4,00E-36	9,00E-36		
						-37,3	-28,4	-30,9	199	145	139	138		
	Ost9901_3:2831	FerrochelataseII		FerrochelataseII	n.a.	SEP3	FerroII	LHC	Ath_FerrII	Cre_Ferrol	Osa_FerrII	Ppa_FerrII	Ferrochelatase II without CB motif, 5´end partial.	Olu_FerrII
						0,31	0,74	4,1	7,00E-142	3,00E-141	6,00E-141	6,00E-140		
						-10,9	-34,8	-44,5	491	489	488	484		
**Physcomitrella patens ssp patens**	Phypa1_1:235408	OHP1.1	Lil2	OHP1	1	OHP1	HLIPa	FerroII	Ppa_OHP1,2	Pta_OHP1__	Osa_OHP1,2	Osa_OHP1,1		Ppa_OHP1.1
JGI Phypa1.1						2,10E-39	2,30E-11	1,60E-07	6,00E-42	3,00E-27	4,00E-27	4,00E-25		
						132,6	39,5	24,1	157	108	108	101		
	Phypa1_1:235406	OPHP1.2	Lil2	OHP1	1	OHP1	HLIPa	SEP	Ppa_OHP1,1	Pta_OHP1__	Osa_OHP1,2	Osa_OHP1,1		Ppa_OHP1.2
						2,90E-41	2,70E-10	2,00E-09	6,00E-42	5,00E-27	7,00E-26	6,00E-25		
						138,8	36	33,1	157	107	103	100		
	Phypa1_1:69369]	OHP2	Lil6	OHP2	1(2*)	OHP2	SEP	HLIPa	Pta_OHP2__	Osa_OHP2__	Ath_OHP2__	Olu_OHP2__		Ppa_OHP2__
						4,50E-38	9,80E-09	3,50E-07	9,00E-44	1,00E-36	2,00E-36	1,00E-23		
						128,2	30,8	15,7	164	140	140	97,8		
	Phypa1_1:235412	SEP1	Lil4	SEP	2	SEP12	SEP	HLIPa	Pta_SEP1__	Osa_SEP1,1	Ath_SEP1__	Osa_SEP1,2		Ppa_SEP1__
						1,40E-29	1,10E-20	4,40E-08	5,00E-28	4,00E-22	4,00E-21	1,00E-12		
						100	70,5	22,5	111	92	89	60,8		
	Phypa1_1:235394	SEP2.1	Lil5	SEP	2	SEP12	SEP	HLIPa	Ppa_SEP2.2	Pta_SEP2__	Osa_SEP2__	Ath_SEP2__		Ppa_SEP2.1
						2,00E-28	2,50E-18	5,20E-06	2,00E-89	3,00E-22	9,00E-20	4,00E-17		
						96,2	62,7	6,7	316	93,6	85,1	76,3		
	Phypa1_1:235393	SEP2.2	Lil5	SEP	2**	SEP12	SEP	HLIPa	Ppa_SEP2.1	Pta_SEP2__	Osa_SEP2__	Ath_SEP2__		Ppa_SEP2.2
						1,30E-28	9,20E-18	6,10E-06	2,00E-89	3,00E-26	9,00E-20	4,00E-17		
						96,9	60,8	6,2	316	106	85,1	76,3		
	Phypa1_1:235409	SEP3.1 (Lil3.1)	Lil3	SEP	2**	SEP3	SEP	OHP2	Ppa_SEP3.3	Ppa_SEP3,2	Ath_SEP3,1	Osa_SEP3__		Ppa_SEP3.1
						6,60E-39	3,70E-26	1,60E-07	5,00E-138	2,00E-83	1,00E-61	8,00E-61		
						131	88,6	10,8	478	297	224	221		
	Phypa1_1:235410	SEP3.2 (Lil3.2)	Lil3	SEP	2**	SEP3	SEP	OHP2	Ppa_SEP3.1	Ppa_SEP3,3	Ath_SEP3,1	Osa_SEP3__		Ppa_SEP3.2
						4,50E-38	2,30E-26	2,70E-06	2,00E-83	3,00E-83	9,00E-63	4,00E-59		
						128,2	89,3	1,1	297	296	228	216		
	Phypa1_1:235411	SEP3.3 (Lil3.3)	Lil3	SEP	2**	SEP3	SEP	OHP2	Ppa_SEP3.1	Ppa_SEP3,2	Osa_SEP3__	Ath_SEP3,1		Ppa_SEP3.3
						1,30E-38	2,70E-25	2,30E-07	5,00E-138	4,00E-84	4,00E-61	6,00E-61		
						130	85,8	9,5	478	299	223	222		

	Phypa1_1:19058	**SEP5**		**SEP**	**n.a.**	**SEP5**	SEP	OHP2	**Pta_SEP5__**	Ath_SEP5__	Osa_SEP5__	Ath_OHP2__	5' end and 3' end not annotated	Ppa_SEP5__
						**4,70E-39**	2,00E-18	5,10E-06	**7,00E-22**	3,00E-20	3,00E-18	1,00E-05		
						**131,5**	63	-1,2	**89,7**	84,3	77,8	36,2		
	Phypa1_1:162400	**SEPx.1**	Lil7	**SEP**	**2**	**SEPxMoss**	SEP	LHL4	**Ppa_SEPx.2_**	Vca_LHL4__	Pta_PSBS1_	Ath_PSBS__		Ppa_SEPx.1_
						**5,10E-35**	1,30E-21	3,00E-11	**4,00E-100**	2,00E-11	3,00E-11	3,00E-11		
						**118,1**	73,6	39,1	**352**	57,4	57	56,6		
	Phypa1_1:161321	**SEPx.2**	Lil7	**SEP**	**2****	**SEPxMoss**	SEP	LHL4	**Ppa_SEPx1_**	Vca_LHL4__	Osa_PsbS,2	Pta_PSBS1_	model manually corrected (erroneous insertion at JGI)	Ppa_SEPx.2_
						**6,50E-25**	9,00E-10	4,30E-06	**4,00E-100**	1,00E-06	1,00E-06	2,00E-06		
						**84,5**	34,2	9,9	**352**	42	42	41,2		
	Phypa1_1:235387	**ELIP******	Lil1	**ELIP**	**3**	**ELIP**	SEP	HLIPa	**Pta_ELIP1_**	Osa_ELIP1_	Osa_ELIP3_	Ath_ELIP1_	multiple copies on scaffold 308 (3x) and scaffold 140 (1x)	na
						**119,7**	57,8	52,4	**2e-025**	5e-024	3e-023	6e-023		
						**1,7e-35**	7,1e-17	2,9e-15	**103**	99,0	96,7	95,5		
	Phypa1_1:116307	**ELIP******	Lil1	**ELIP**	**3**	**ELIP**	SEP	HLIPa	**Pta_ELIP1_**	Ath_ELIP1_	Osa_ELIP3_	Osa_ELIP1_	5´end partial; two copies on scaffold 15	na
						**119,1**	56,6	52,4	**8e-026**	2e-023	3e-023	3e-023		
						**2,5e-35**	1,6e-16	2,9e-15	**104**	96,3	95,9	95,9		
	Phypa1_1:235386	**ELIP**	Lil1	**ELIP**	**3**	**ELIP**	SEP	HLIPa	**Pta_ELIP1_**	Ath_ELIP1_	Osa_ELIP3_	Osa_ELIP1_		na
						**119,4**	56,3	44,9	**3e-024**	5e-023	8e-023	1e-022		
						**2e-35**	2e-16	5,7e-13	**99,8**	95,9	95,1	94,4		
	Phypa1_1:235391	**ELIP**	Lil1	**ELIP**	**3**	**ELIP**	SEP	SEPxGlauco	**Osa_ELIP3_**	Ath_ELIP1_	Osa_ELIP1_	Pta_ELIP1_		na
						**111,2**	66,5	45,1	**4e-023**	5e-023	1e-022	2e-022		
						**5,9e-33**	1,8e-19	4,9e-13	**96,3**	95,9	94,7	93,6		
	Phypa1_1:152573	**ELIP**	Lil1	**ELIP**	**3**	**ELIP**	SEP	SEPxGlauco	**Osa_ELIP3_**	Osa_ELIP1_	Ath_ELIP1_	Pta_ELIP3_		na
						**109,9**	66,5	46,5	**2e-022**	3e-022	5e-022	5e-022		
						**1,5e-32**	1,8e-19	1,8e-13	**93,6**	92,8	92,0	92,0		
	Phypa1_1:235392	**ELIP**	Lil1	**ELIP**	**3**	**ELIP**	SEP	SEPxGlauco	**Osa_ELIP3_**	Osa_ELIP1_	Pta_ELIP3_	Osa_ELIP2_		na
						**103,3**	62,3	39,0	**1e-023**	1e-022	3e-021	6e-021		
						**1,4e-30**	3,2e-18	3,2e-11	**98,2**	94,7	89,7	89,0		
	Phypa1_1:164842	**ELIP**	Lil1	**ELIP**	**3**	**ELIP**	SEP	HLIPa	**Osa_ELIP1_**	Osa_ELIP3_	Osa_ELIP2_	Pta_ELIP3_		na
						**115,1**	68,9	66,1	**1e-025**	2e-025	1e-024	9e-023		
						**4,1e-34**	3,2e-20	2,3e-19	**105**	104	102	95,5		
	Phypa1_1:93237	**ELIP**	Lil1	**ELIP**	**3**	**ELIP**	SEP	HLIPa	**Pta_ELIP1_**	Pta_ELIP3_	Osa_ELIP1_	Osa_ELIP3_	manually corrected	na
						**110,0**	66,8	51,4	**2e-023**	2e-022	9e-022	2e-021		
						**1,4e-32**	1,4e-19	6e-15	**97,4**	93,6	91,7	90,5		
	Phypa1_1:118582	**ELIP**	Lil1	**ELIP**	**3**	**ELIP**	SEP	HLIPa	**Ath_ELIP2_**	Pta_ELIP1_	Ath_ELIP1_	Pta_ELIP3_	5´end partial	na
						**125,9**	71,8	56,0	**2e-026**	3e-024	3e-023	7e-023		
						**2,3e-37**	4,4e-21	2,5e-16	**106**	99,0	95,5	94,4		
	Phypa1_1:196099	**ELIP******	Lil1	**ELIP**	**3**	**ELIP**	SEP	HLIPa	**Osa_ELIP1_**	Osa_ELIP3_	Osa_ELIP2_	Pta_ELIP1_	5´end partial; two copies on scaffold 255	na
						**118,2**	76,8	54,2	**2e-026**	7e-026	7e-026	1e-023		
						**4,7e-35**	1,4e-22	8,5e-16	**105**	104	104	96,7		
	Phypa1_1:235390	**ELIP**	Lil1	**ELIP**	**3**	**HLIPa**	SEP	HLIPb	**Pta_ELIP3_**	Osa_ELIP1_	Osa_ELIP2_	Pta_ELIP1_	CB motifs rather divergent; 5´end partial	na
						**34,7**	34,4	14,7	**2e-017**	2e-016	5e-016	1e-015		
						**6,5e-10**	8,2e-10	7,3e-06	**75,9**	72,4	71,2	69,7		
	Phypa1_1:55357	**ELIP******	Lil1	**ELIP**	**3**	**ELIP**	SEP	SEPxGlauco	**Osa_ELIP3_**	Osa_ELIP1_	Osa_ELIP2_	Ath_ELIP1_	two copies on scaffold 10	na
						**109,5**	67,4	48,9	**2e-024**	4e-024	2e-022	9e-022		
						**2e-32**	9,4e-20	3,3e-14	**100**	99,4	94,0	91,7		
	Phypa1_1:235423	**ELIP**	Lil1	**ELIP**	**3**	**ELIP**	SEP	HLIPa	**Pta_ELIP2_**	Pta_ELIP1_	Ath_ELIP1_	Cre_ELIP7_		na
						**93,1**	46,8	40,1	**3e-014**	1e-012	2e-012	5e-012		
						**1,7e-27**	1,5e-13	1,6e-11	**66,6**	61,6	60,5	59,3		
	Phypa1_1:235377	**PSBS**	CP22	**PSBS**	**4**	**PSBS**	LHL4	SEPxMoss	**Osa_PsbS,2**	Pta_PSBS1_	Osa_PsbS,1	Ath_PSBS__		Ppa_PSBS__
						**4,00E-58**	9,10E-27	3,90E-23	**5,00E-99**	6,00E-95	8,00E-92	2,00E-90		
						**194,8**	90,7	78,6	**348**	335	324	320		
	Phypa1_1:235459	**Rieske-like CAB**		**Rieske-like CAB**	**2**	**RieskeCB**	SEP	HLIPa	**Ath_Rieske**	Osa_Rieske	Pta_Rieske	Cre_Rieske		Ppa_Rieske
						**5,20E-42**	1,70E-15	5,60E-14	**6,00E-80**	5,00E-77	5,00E-50	5,00E-37		
						**141,3**	53,2	48,2	**285**	275	186	142		
	Phypa1_1:31862	**FerrochelataseII**		**FerrochelataseII**	**1**	**FerroII**	HLIPa	SEP	**Osa_FerrII**	Ath_FerrII	Cre_FerroI	Olu_FerrII	Ferrochelatase II with CB motif, 5´end not annotated	Ppa_FerrII
						**5,40E-50**	3,00E-19	4,30E-13	**0**	0	9,00E-170	7,00E-140		
						**167,8**	65,7	45,3	**702**	682	584	484		
**Pinus taeda**	gi_49012632_	**OHP1**	Lil2	**OHP1**	**1**	**OHP1**	HLIPa	SEP	**Osa_OHP1,2**	Osa_OHP1,1	Ath_OHP1__	Ppa_OHP1,1		Pta_OHP1__
NCBI EST	gb_CO201457_1_					**8,00E-38**	7,50E-09	9,40E-07	**4,00E-33**	4,00E-30	3,00E-29	3,00E-27		
						**127,4**	28,3	22,8	**127**	117	115	108		
	gi_74157520_	**OHP2**	Lil6	**OHP2**	**1**	**OHP2**	SEP	HLIPa	**Osa_OHP2__**	Ppa_OHP2__	Ath_OHP2__	Olu_OHP2__		Pta_OHP2__
	gb_DT627452_1					**1,10E-35**	4,20E-12	7,80E-09	**2,00E-44**	8,00E-44	4,00E-43	3,00E-23		
						**120,3**	41,9	28,2	**166**	164	162	95,9		
	gi_66746600_	**SEP1**	Lil4	**SEP**	**2**	**SEP12**	SEP	HLIPa	**Ppa_SEP1__**	Osa_SEP1,1	Ath_SEP1__	Osa_SEP1,2		Pta_SEP1__
	gb_DR024230_1					**1,20E-23**	2,40E-15	2,10E-07	**5,00E-28**	1,00E-25	2,00E-24	2,00E-15		
						**80,4**	52,7	17,2	**111**	103	99,8	69,7		
	gi_48949565_	**SEP2**	Lil5	**SEP**	**2**	**SEP12**	SEP	HLIPa	**Osa_SEP2__**	Ath_SEP2__	Ppa_SEP2,2	Ppa_SEP2,1		Pta_SEP2__
	gb_CO176693					**3,70E-19**	3,70E-14	2,30E-07	**1,00E-33**	6,00E-30	2,00E-26	2,00E-22		
						**65,4**	48,8	17	**130**	118	106	93,6		
	gi_49010503_	**SEP3**	Lil3	**SEP**	**n.a.**	**SEP3**	SEP	ELIP	**Ath_SEP3,1**	Ath_SEP3,2	Osa_SEP3__	Ppa_SEP3,2	5' end and 3' end partial	Pta_SEP3__
	gb_CO199328_1					**6,20E-24**	5,20E-12	0,0066	**6,00E-20**	1,00E-18	3,00E-18	9,00E-17		
						**81,3**	41,6	-13,6	**83,2**	79	77,8	72,8		
	gi_70776652_	**SEP4**		**SEP**	**2**	**SEP4**	SEP	HLIPa	**Ath_SEP4__**	Osa_SEP4__	Glo_HLIP2_	Cpa_SEPx.1_		Pta_SEP4__
	gb_DR688176_					**1,20E-33**	1,80E-14	7,60E-12	**4,00E-40**	1,00E-34	1,00E-07	2,00E-06		
						**113,6**	49,8	41,1	**152**	134	44,7	40,4		
	gi_67556374_	**SEP5**		**SEP**	**1(2)**	**SEP5**	SEP	OHP2	**Osa_SEP5__**	Ath_SEP5__	Ppa_SEP5__	Vca_LHL4__	CB motif in first helix, possibly additional 3´terminal second helix or membrane anchor	Pta_SEP5__
	gb_DR097019_1_					**4,10E-33**	1,50E-19	8,80E-07	**1,00E-25**	5,00E-24	1,00E-21	5,00E-08		
						**111,7**	66,7	4,9	**102**	97,4	89,7	44,3		
	gi_66980661_	**ELIP1******	Lil1	**ELIP**	**3**	**ELIP**	SEP	HLIPa	**Pta_ELIP2_**	Ath_ELIP1_	Ath_ELIP2_	Pta_ELIP3_		Pta_ELIP1_
	gb_DR057094_1_					**2,20E-33**	9,50E-18	2,00E-13	**5,00E-66**	6,00E-49	1,00E-47	5,00E-40		
						**112,7**	60,7	46,4	**238**	181	177	152		
	gi_34489189_	**ELIP2******	Lil1	**ELIP**	**3**	**ELIP**	SEP	HLIPa	**Pta_ELIP1_**	Ath_ELIP1_	Ath_ELIP2_	Pta_ELIP3_		Pta_ELIP2_
	gb_CF471817_1					**5,20E-34**	2,10E-20	2,40E-13	**4,00E-66**	2,00E-41	6,00E-39	2,00E-38		
						**114,7**	69,5	46,1	**238**	156	148	146		
	gi_34489018_	**ELIP3******	Lil1	**ELIP**	**3**	**ELIP**	SEP	HLIPa	**Pta_ELIP1_**	Pta_ELIP2_	Ath_ELIP1_	Ath_ELIP2_		Pta_ELIP3_
	gb_CF471646_1_					**1,10E-21**	2,40E-15	7,80E-11	**2,00E-40**	1,00E-38	5,00E-38	3,00E-36		
						**73,8**	52,8	37,8	**152**	146	144	138		
	gi_67551620_	**PSBS.1**	CP22	**PSBS**	**4**	**PSBS**	SEPxMoss	LHL4	**Pta_PSBS2_**	Osa_PsbS,2	Ath_PSBS__	Osa_PsbS,1		Pta_PSBS1_
	gb_DR092647_1_					**1,20E-60**	2,40E-25	4,60E-25	**1,00E-110**	9,00E-101	1,00E-99	7,00E-98		
						**203,3**	86	85	**387**	354	350	344		
	gi_48945925_	**PSBS.2**	CP22	**PSBS**	**4**	**PSBS**	LHL4	SEPxMoss	**Pta_PSBS1_**	Ath_PSBS__	Osa_PsbS,2	Osa_PsbS,1		Pta_PSBS2_
	gb_CO173053_1					**6,90E-60**	1,70E-27	2,90E-25	**7,00E-111**	9,00E-96	2,00E-92	1,00E-91		
						**200,7**	93,1	85,7	**387**	337	326	323		
	gi_49442568_	**Rieske-like CAB**		**Rieske-like CAB**	**1**	**RieskeCB**	SEP	HLIPa	**Ath_Rieske**	Osa_Rieske	Ppa_Rieske	Cre_Rieske		Pta_Rieske
	gb_CO361251_1					**2,40E-36**	5,10E-10	1,00E-07	**2,00E-68**	5,00E-67	2,00E-50	5,00E-24		
						**122,5**	35	19,7	**246**	241	186	98,6		
	gi_34491765_	**FerrochelataseII**		**FerrochelataseII**	**n.a.**	**FerroII**	SEP5	SEP4	**Osa_FerrII**	Ath_FerrII	Ppa_FerrII	Cre_FerroI	5' end and 3' end partial; no CB motif but probably due to missing EST data	Pta_FerrII
	gb_CF474393_1_					**0,00014**	5,3	5,6	**1,00E-56**	3,00E-55	2,00E-54	1,00E-40		
						**-1,9**	-34,2	-39	**206**	201	199	152		
**Arabidopsis thaliana**	At5g02120	**Ohp1**	Lil2	**OHP1**	**1**	**OHP1**	HLIPa	SEP	**Osa_OHP1,2**	Osa_OHP1,1	Pta_OHP1__	Ppa_OHP1,2		Ath_OHP1__
TAIR						**9,80E-41**	4,90E-12	3,30E-08	**2,00E-31**	6,00E-30	3,00E-29	7,00E-25		
						**137,1**	41,7	29	**122**	117	115	100		
	At1g34000	**Ohp2**	Lil6	**OHP2**	**1(2*)**	**OHP2**	SEP	HLIPa	**Osa_OHP2__**	Pta_OHP2__	Ppa_OHP2__	Olu_OHP2__		Ath_OHP2__
						**1,00E-38**	2,30E-12	8,00E-08	**4,00E-46**	4,00E-43	2,00E-36	5,00E-21		
						**130,4**	42,9	20,5	**172**	162	140	88,6		
	At4g34190	**SEP1**	Lil4	**SEP**	**2**	**SEP12**	SEP	LHL4	**Pta_SEP1__**	Osa_SEP1,1	Ppa_SEP1__	Osa_SEP1,2		Ath_SEP1__

						1,90E-30	2,10E-21	7,70E-07	2,00E-24	4,00E-24	3,00E-21	4,00E-20		
						102,9	72,8	15,3	99,8	98,6	89	85,5		
	At2g21970	SEP2	Lil5	SEP	2*	SEP12	SEP	HLIPa	Osa_SEP2__	Pta_SEP2__	Ppa_SEP2,2	Ppa_SEP2,1		Ath_SEP2__
						7,20E-31	1,40E-19	1,90E-05	2,00E-48	6,00E-30	3,00E-17	3,00E-17		
						104,3	66,8	2,4	179	118	76,3	76,3		
	At4g17600	SEP3.1 (Lil3.1)	Lil3	SEP	2	SEP3	SEP	HLIPa	Ath_SEP3,2	Osa_SEP3__	Ppa_SEP3,2	Ppa_SEP3,1		Ath_SEP3.1
						3,80E-40	2,00E-25	2,10E-07	5,00E-115	1,00E-78	6,00E-63	1,00E-61		
						135,1	86,2	17,3	401	281	228	224		
	At5g47110	SEP3.2 (Lil3.2)	Lil3	SEP	2	SEP3	SEP	HLIPa	Ath_SEP3,1	Osa_SEP3__	Ppa_SEP3,3	Ppa_SEP3,1		Ath_SEP3.2
						6,30E-40	2,40E-25	1,10E-07	5,00E-115	7,00E-79	6,00E-60	6,00E-60		
						134,4	86	19,5	401	281	218	218		
	At3g12345	SEP4		SEP	2	SEP4	SEP	HLIPa	Pta_SEP4__	Osa_SEP4__	Glo_HLIP2_	Ath_ELIP1_	one of several new LHC-like sequences in Arabidopsis thaliana	Ath_SEP4__
						7,20E-43	6,30E-21	2,70E-09	3,00E-40	3,00E-32	2,00E-08	1,00E-07		
						144,2	71,3	31,7	152	126	47	44,3		
	At4g28025	SEP5		SEP	1(2)	SEP5	SEP	HLIPa	Osa_SEP5__	Pta_SEP5__	Ppa_SEP5__	Vca_LHL4__	CB motif in first helix, possibly additional 3´terminal second helix or membrane anchor	Ath_SEP5__
						4,90E-39	1,10E-20	3,00E-05	3,00E-26	1,00E-23	9,00E-20	2,00E-07	one of several new LHC-like sequences in Arabidopsis thaliana	
						131,4	70,4	0,9	105	97,4	84,3	43,5		
	At3g22840	Elip1	Lil1	ELIP	3	ELIP	SEP	HLIPa	Ath_ELIP2_	Pta_ELIP1_	Osa_ELIP1_	Osa_ELIP2_		Ath_ELIP1_
						3,40E-33	1,60E-18	9,90E-12	4,00E-78	5,00E-49	7,00E-46	2,00E-45		
						112	63,2	40,7	278	181	171	170		
	At4g14690	Elip2	Lil1	ELIP	3	ELIP	SEP	HLIPa	Ath_ELIP1_	Pta_ELIP1_	Osa_ELIP2_	Osa_ELIP1_		Ath_ELIP2_
						3,20E-33	3,30E-22	5,00E-15	4,00E-78	1,00E-47	8,00E-45	5,00E-44		
						112,1	75,5	51,7	278	177	167	165		
	At1g44575.1	PSBS	CP22	PSBS	4	PSBS	SEPxMoss	LHL4	Osa_PsbS,2	Osa_PsbS,1	Pta_PSBS1_	Pta_PSBS2_		Ath_PSBS__
						2,60E-61	1,30E-25	1,40E-25	2,00E-101	7,00E-101	1,00E-99	1,00E-95		
						205,4	86,8	86,8	356	354	350	337		
	At1g71500	Rieske-like CAB		Rieske-like CAB	1	RieskeCB	HLIPa	SEP	Osa_Rieske	Ppa_Rieske	Pta_Rieske	Cre_Rieske	one of several new LHC-like sequences in Arabidopsis thaliana	Ath_Rieske
						1,60E-41	5,00E-10	8,20E-10	7,00E-99	6,00E-80	4,00E-68	2,00E-38		
						139,7	35,1	34,4	348	285	246	147		
	At2g30390	FerrochelataseII		FerrochelataseII	1	FerroII	HLIPa	SEP	Osa_FerrII	Ppa_FerrII	Cre_FerroI	Olu_FerrII		Ath_FerrII
						4,70E-51	5,10E-19	2,50E-14	0	0	3,00E-164	1,00E-141		
						171,4	64,9	49,4	739	682	566	491		
**Oryza sativa ssp. japonica**	Os12g29570	Ohp1.1	Lil2	OHP1	1	OHP1	HLIPa	SEP	Osa_OHP1,2	Pta_OHP1__	Ath_OHP1__	Ppa_OHP1,1		Osa_OHP1.1
TIGR						1,30E-40	1,70E-09	1,60E-06	5,00E-35	5,00E-30	6,00E-30	3,00E-25		
						136,6	33,3	20,8	134	117	117	101		
	Os05g22730	Ohp1.2	Lil2	OHP1	1	OHP1	HLIPa	SEP	Osa_OHP1,1	Pta_OHP1__	Ath_OHP1__	Ppa_OHP1,1		Osa_OHP1.2
						2,90E-42	1,30E-11	5,00E-07	5,00E-35	4,00E-33	2,00E-31	4,00E-27		
						142,2	40,4	25,1	134	127	122	108		
	Os01g40710	Ohp2	Lil6	OHP2	1(2*)	OHP2	SEP	SEPxGlauco	Ath_OHP2__	Pta_OHP2__	Ppa_OHP2__	Olu_OHP2__		Osa_OHP2__
						1,10E-38	5,40E-09	1,00E-07	4,00E-46	2,00E-44	1,00E-36	4,00E-20		
						130,2	31,6	24,9	172	166	140	85,9		
	Os10g25570	SEP1.1	Lil4	SEP	2	SEP12	SEP	LHL4	Pta_SEP1__	Ath_SEP1__	Osa_SEP1,2	Ppa_SEP1__		Osa_SEP1.1
						9,50E-30	1,50E-19	4,20E-06	9,00E-26	4,00E-24	2,00E-22	4,00E-22		
						100,6	66,7	10	103	98,6	93,2	92		
	Os11g40600	SEP1.2	Lil4	SEP	2**	SEP12	SEP	LHL4	Osa_SEP1,1	Ath_SEP1__	Pta_SEP1__	Ppa_SEP1__		Osa_SEP1.2
						7,10E-26	1,90E-16	1,20E-05	2,00E-22	3,00E-20	2,00E-15	8,00E-13		
						87,7	56,4	6,8	93,2	85,5	69,7	60,8		
	Os04g54630	SEP2	Lil5	SEP	2	SEP12	SEP	HLIPa	Ath_SEP2__	Pta_SEP2__	Ppa_SEP2,2	Ppa_SEP2,1		Osa_SEP2__
						1,50E-31	1,50E-20	1,90E-06	2,00E-48	1,00E-33	7,00E-20	7,00E-20		
						106,5	70,1	10	179	130	85,1	85,1		
	Os02g03330	SEP3	Lil3	SEP	2	SEP3	SEP	HLIPa	Ath_SEP3,2	Ath_SEP3,1	Ppa_SEP3,3	Ppa_SEP3,1		Osa_SEP3__
						1,30E-40	1,60E-26	7,20E-08	7,00E-79	9,00E-79	3,00E-61	6,00E-61		
						136,7	89,8	20,8	281	281	223	221		
	Os06g28950	SEP4		SEP	2	SEP4	SEP	HLIPa	Pta_SEP4__	Ath_SEP4__	Glo_HLIP2_	Olu_OHP1__		Osa_SEP4__
						6,50E-39	1,80E-15	4,30E-09	1,00E-34	3,00E-32	2,00E-07	1,00E-06		
						131	53,2	30,2	134	126	44,3	41,6		
	Os02g39730	SEP5		SEP	2	SEP5	SEP	OHP2	Ath_SEP5__	Pta_SEP5__	Ppa_SEP5__	Gno_SEPx.6_	CB motif in first helix, likely additional 3´terminal second helix or membrane anchor	Osa_SEP5__
						3,10E-38	2,30E-21	1,60E-07	3,00E-26	2,00E-25	8,00E-18	2,00E-08		
						128,8	72,7	10,7	105	102	77,8	46,6		
	Os07g08150	ELIP1	Lil1	ELIP	3	ELIP	SEP	SEPxGlauco	Osa_ELIP3_	Osa_ELIP2_	Ath_ELIP1_	Ath_ELIP2_	ELIP number newly assigned	Osa_ELIP1_
						2,30E-33	1,80E-23	4,00E-12	5,00E-93	5,00E-93	8,00E-46	5,00E-44		
						112,6	79,7	42,1	328	328	171	165		
	Os01g14410	ELIP2	Lil1	ELIP	3	ELIP	SEP	HLIPa	Osa_ELIP1_	Osa_ELIP3_	Ath_ELIP1_	Ath_ELIP2_	ELIP number newly assigned	Osa_ELIP2_
						5,20E-34	2,30E-22	1,70E-12	5,00E-93	6,00E-93	2,00E-45	9,00E-45		
						114,7	76	43,3	328	327	170	167		
	Os07g08160	ELIP3	Lil1	ELIP	3	ELIP	SEP	HLIPa	Osa_ELIP1_	Osa_ELIP2_	Ath_ELIP1_	Ath_ELIP2_	ELIP number newly assigned	Osa_ELIP3_
						1,50E-33	7,50E-22	9,30E-12	5,00E-93	6,00E-93	2,00E-43	3,00E-43		
						113,2	74,4	40,8	328	327	163	162		
	Os04g59440	PSBS.1	CP22	PSBS	4	PSBS	SEPxMoss	LHL4	Osa_PsbS,2	Ath_PSBS__	Pta_PSBS1_	Ppa_PSBS__		Osa_PsbS.1
						1,10E-59	8,20E-24	1,60E-22	8,00E-102	7,00E-101	7,00E-98	7,00E-92		
						200	80,9	76,6	357	354	344	324		
	Os01g64960	PSBS.2	CP22	PSBS	4	PSBS	LHL4	SEPxMoss	Osa_PsbS,1	Ath_PSBS__	Pta_PSBS1_	Ppa_PSBS__		Osa_PsbS.2
						1,10E-59	6,90E-27	8,30E-24	8,00E-102	2,00E-101	9,00E-101	5,00E-99		
						200,1	91,1	80,8	357	356	354	348		
	Os11g13850	Rieske-like CAB		Rieske-like CAB	1	RieskeCB	HLIPa	SEP	Ath_Rieske	Ppa_Rieske	Pta_Rieske	Olu_Rieske		Osa_Rieske
						4,80E-41	2,50E-09	2,90E-09	7,00E-99	4,00E-77	9,00E-67	5,00E-38		
						138,1	31,9	32,5	348	275	241	145		
	Os05g29760	FerrochelataseII		FerrochelataseII	1	FerroII	HLIPa	SEP	Ath_FerrII	Ppa_FerrII	Cre_FerroI	Olu_FerrII		Osa_FerrII
						1,40E-50	1,50E-17	2,10E-14	0	0	2,00E-163	8,00E-141		
						169,8	60	49,6	739	702	563	488		

*** name of HMM profile	comment
HLIPa	HMM profile based on first HLIP seed alignment
HLIPb	HMM profile based on second HLIP seed alignment
OHP1	HMM profile of OHP1
OHP2	HMM profile of OHP2
SEP12	HMM profile of SEP1 and SEP2
SEP3	HMM profile of SEP3
SEP4	HMM profile of SEP4
SEP5	HMM profile of SEP5
SEPxGlauco	HMM profile of glaucophyte SEPx
SEPxMoss	HMM profile of SEPx in Physcomitrella and Selaginella
SEP	HMM profile of all typical two-helix SEP sequences
RedCAP	HMM profile of RedCAP
ELIP	HMM profile of ELIP
LHL4	HMM profile of Lhl4
PSBS	HMM profile of PSBS
RieskeCB	HMM profile of Rieske-like CAB fusion proteins
FerroII	HMM profile of Ferrochelatase II
LHC	HMM profile of LHC including all major LHC subfamilies

References

Alboresi, A., S. Caffarri, F. Nogue, R. Bassi, and T. Morosinotto. 2008. In silico and biochemical analysis of Physcomitrella patens photosynthetic antenna: identification of subunits which evolved upon land adaptation. PLoS ONE 3:e2033.

Elrad, D., and A. R. Grossman. 2004. A genome's-eye view of the light-harvesting polypeptides of Chlamydomonas reinhardtii. Curr Genet 45:61-75.

Funk, C., and W. Vermaas. 1999. A cyanobacterial gene family coding for single-helix proteins resembling part of the light-harvesting proteins from higher plants. Biochemistry 38:9397-9404.

Jansson, S. 1999. A guide to the Lhc genes and their relatives in Arabidopsis/IT>. Trends Plant Sci 4:236-240.

Klimmek, F., A. Sjodin, C. Noutsos, D. Leister, and S. Jansson. 2006. Abundantly and rarely expressed Lhc protein genes exhibit distinct regulation patterns in plants. Plant Physiol 140:793-804.

Rensing, S. A., D. Lang, A. D. Zimmer et al. 2008. The Physcomitrella genome reveals evolutionary insights into the conquest of land by plants. Science 319:64-69.

Teramoto, H., T. Itoh, and T. A. Ono. 2004. High-intensity-light-dependent and transient expression of new genes encoding distant relatives of light-harvesting chlorophyll-a/b proteins in Chlamydomonas reinhardtii. Plant Cell Physiol 45:1221-1232.

Table S2

Identified Sequences of the Extended LHC Protein Superfamily and Their Classification Exemplified for the Glaucophyte *C. paradoxa*

Proposed name	Gene model (Database)	Classified as	Number of TM helices	Best HMM model	Best local BLASTP	Alternative names	Comments
HLIP	P48367 (Uniprot)	HLIP	1	HLIPa 2,70E-28 95,8	Gsu HLIP 6,00E-20 83,2	ycf17	Complete CDS, cyanelle-encoded
OHP1-like.1	BK006744 (NCBI_TPA)	OHP1-like	1	HLIPa 3,00E-15 52,4	Cpa OHP1-like.2 1,00E-49 183	-	5´end partial but TP predicted, nuclear-encoded
OHP1-like.2	BK006745 (NCBI_TPA)	OHP1-like	1	HLIPa 2,90E-16 55,8	Cpa OHP1-like1 2,00E-49 183	-	5´end partial but TP predicted, nuclear-encoded

OHP2	BK006746<br>(NCBI_TPA)	OHP2	1	OHP2<br>9,80E-35<br>117,1	Gsu OHP2<br>8,00E-12<br>58,5	Lil6	Complete CDS
SEPx.1	BK006747	SEP	2	SEP<br>1,60E-18<br>63,3	Gno SEPx.1<br>2,00E-20<br>87	-	Complete CDS
SEPx.2	BK006748	SEP	2	SEPxGlauco<br>2,00E-36<br>122,7	Gno SEPx.2<br>1,00E-24<br>100	-	Complete CDS
Ferrochelatase II	EC660148<br>& EC656004<br>(NCBI EST)	Ferrochelatase II	2	HLIPa<br>5,90E-10<br>34,8	Ana Ferro<br>9,00E-102<br>357	-	5´end partial,<br>intact CB motif

NOTE.—These sequences except the plastid HLIP (ycf17) were newly identified based on EST data and deposited at NCBI_TPA under the given accession numbers. Three-letter abbreviations are used as follows: Gsu (*Galdieria sulphuraria*), Cpa (*Cyanophora paradoxa*), Gno (*Glaucocystis nostochinearum*), and Ana (*Anabena* sp. PCC 7120). CB, chlorophyll-binding motif; CDS, coding sequence; TM, transmembrane motif; TP, transit peptide.
